# Electro-optic spatial light modulator from an engineered organic layer

**DOI:** 10.1038/s41467-021-26035-y

**Published:** 2021-10-11

**Authors:** Ileana-Cristina Benea-Chelmus, Maryna L. Meretska, Delwin L. Elder, Michele Tamagnone, Larry R. Dalton, Federico Capasso

**Affiliations:** 1 Harvard John A. Paulson School of Engineering and Applied Sciences, Harvard University, Cambridge, MA USA; 2 Department of Chemistry, University of Washington, Seattle, WA USA

**Keywords:** Nanophotonics and plasmonics, Optoelectronic devices and components

## Abstract

Tailored nanostructures provide at-will control over the properties of light, with applications in imaging and spectroscopy. Active photonics can further open new avenues in remote monitoring, virtual or augmented reality and time-resolved sensing. Nanomaterials with *χ*^(2)^ nonlinearities achieve highest switching speeds. Current demonstrations typically require a trade-off: they either rely on traditional *χ*^(2)^ materials, which have low non-linearities, or on application-specific quantum well heterostructures that exhibit a high *χ*^(2)^ in a narrow band. Here, we show that a thin film of organic electro-optic molecules JRD1 in polymethylmethacrylate combines desired merits for active free-space optics: broadband record-high nonlinearity (10-100 times higher than traditional materials at wavelengths 1100-1600 nm), a custom-tailored nonlinear tensor at the nanoscale, and engineered optical and electronic responses. We demonstrate a tuning of optical resonances by Δ*λ* = 11 nm at DC voltages and a modulation of the transmitted intensity up to 40%, at speeds up to 50 MHz. We realize 2 × 2 single- and 1 × 5 multi-color spatial light modulators. We demonstrate their potential for imaging and remote sensing. The compatibility with compact laser diodes, the achieved millimeter size and the low power consumption are further key features for laser ranging or reconfigurable optics.

## Abstract (web summary)

Spatial light modulators (SLM) provide tailored light fields for many applications. Here, the authors present an SLM device based on an organic electro-optic material that manipulates the properties of individual pixels by electronic signals at speeds up to 50 MHz.

## Introduction

Recent advances in both traditional and novel electro-optic materials that exhibit a *χ*^(2)^ nonlinearity have resulted into an unprecedented richness of active photonic devices, that find applications in communications^1–3^, electric field metrology^4,5^, dynamic beam steering^6^, and quantum science^7^. Multi-pixel, large-area spatial light modulators (SLMs) are a prerequisite to achieve massively parallel dynamic and reconfigurable control over the diverse properties of light. They promise to revolutionize applications in industry and fundamental science. In reconfigurable photonics, multipixel optical components can control light on-demand, but they need to be compact and consume minimal power per pixel. In massively parallel remote sensing^8^, high-speed SLMs can generate parallel optical channels that scan the environment in three dimensions, but operation at high speeds is mandatory to resolve small changes in the position and velocity of objects within a short timeframe. In the area of ultrafast optics, SLMs can manipulate femtosecond pulses through pulse-pickers, but must accommodate a broad optical bandwidth and switching speeds commensurate with the repetition rate of these lasers, typically around few MHz to GHz. In fundamental science, SLMs play a crucial role e.g., in the sorting and reconfiguration of cold atoms for quantum simulation^9^, and reaching higher speeds can access entirely new physics. In industry applications, such as self-driving cars^10^, high-speed SLMs can monitor a scene if they are compatible with low-power electronic circuits, chip-based laser diodes that typically have a broad linewidth and low foot-print packaging.

Among all existing SLM technologies, SLMs that employ electric tuning enabled by *χ*^(2)^ materials are outstanding candidates to reach high speeds in parallel multipixel architectures. They are intrinsically compatible with massively parallel radio-frequency (RF) electronic circuits that promise to deliver the necessary electrical controls by hybrid electronic-optical integration. In this case, the SLM is essentially a two-dimensional array of free-space electro-optic modulators. Commercial SLMs instead use bulky digital micro-mirror arrays^11^ or liquid crystals^12^, which not only limit them to around 10 kHz but also do not employ nanostructures. Few state-of-the art SLM demonstrations address the quest for ever-higher speed by making use of narrow-band surface plasmon resonances in rather complex prism-based schemes using *χ*^(2)^ nonlinear materials^13^, but the nano-scale engineering of the individual pixels remains unexplored and the employed nonlinearity is rather low. Flat lens technology around metasurfaces^14^ is a powerful platform that permits engineering of the properties of light at a sub-wavelength scale in an extremely compact device. Static single-pixel metasurfaces have addressed multiple needs (focussing, polarization control^15^, or correcting optics^16^) or multipixel metasurfaces (next-generation biodevices^17^). Consequently, an ideal platform for high-speed SLMs combines high-performance *χ*^(2)^ materials with nanostructures. Current demonstrations of monolithic metasurfaces from *χ*^(2)^ materials typically trade off an operation around a sharp resonance in materials with moderate but broadband *χ*^(2)^ effect e.g., in lithium niobate^18,19^ or colloidal nonlinear nanocubes^20^ against an efficient *χ*^(2)^ effect in application-specific engineered III–V heterostructures, which is however narrowband and located around its bandgap^6^. While many demonstrations are single-pixel, few exciting multifunctional metasurfaces with multiple electronic controls have emerged experimentally^6,21,22^ and theoretically^23^. Important to mention are also metasurfaces based on transparent conductive oxides^22,24–26^ that have enabled SLMs recently^27^, gate-tunable low-dimensional materials^28^ or phase change materials such as e.g., GST^29–33^ that are excellent candidates in scenarios, where high switching speeds are not required. Microelectromechanical^34,35^ or thermo-optically controlled^36^ systems are ideal for low speed applications that do not require pixel-level control.

Custom-engineered organic nonlinear molecules instead overcome this bandwidth-nonlinearity limitation since they have electro-optic coefficients that are 10–100 times higher than standard materials (up to *r*_33_ = 560 pmV^−1^ in bulk layers and 200 pmV^−1^ in nanoplasmonic gaps of gold electrodes^37^), over the entire band from 1100 to 1600 nm (see Supplementary Note 7). They are anticipated to deliver high-end SLMs, owing to their terahertz-compatible bandwidth (up to 2.5 THz^38^), high index of refraction and recently reported long-term thermal and chemical stability^39^. Only little work studies such molecules beyond silicon photonic circuits (where they reach highest electro-optic couplings^4^ and integration with complementary metal-oxide-semiconductor (CMOS) electronics^40^) for large-area electro-optic modulators, even in the single pixel case^41,42^. The only SLMs demonstrated so far to the best of our knowledge employ vertical Fabry-Perot geometries that do not exploit nano-engineering, and force an operation with the much lower *r*_13_ electro-optic coefficient^43^.

In this work, we experimentally demonstrate that a single layer of organic non-linear material (here JRD1 mixed with PMMA) enables custom-tailored engineering of each pixel of an SLM while, at the same time, providing a high modulation efficiency through *r*_33_, compatibility with broadband lasers, linearity to driving voltage, low power consumption, and high speed. This stems from our ability to manipulate the tensorial properties of the nonlinearity (magnitude and orientation) of the thin film at the nanoscale at will by electric field poling^44^, and influence the electric current per pixel by mixing of JRD1 with PMMA.

## Results

### Electro-optic spatial light modulator concept

**Fig. 1:**
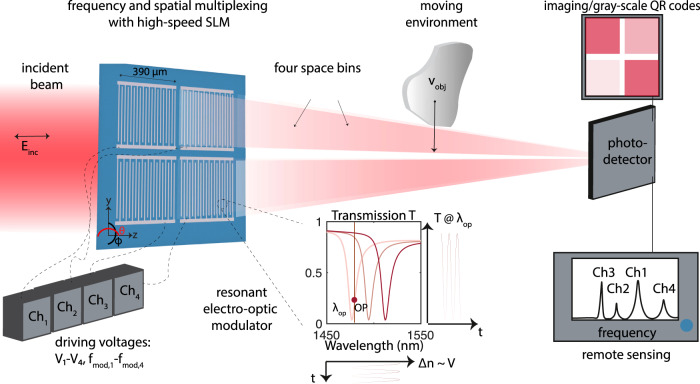
Large area, high-speed, electro-optic spatial light modulator for massively parallel imaging or remote sensing. A beam of light *E*_inc_ is incident onto an array of *n* × *n* electro-optic modulators that are controlled by a set of driving voltages *V*_n_ at radio-frequencies ${f}_{{{{{{{{\rm{mod}}}}}}}},{{{{{{{\rm{n}}}}}}}}}$. The modulators are resonant by design and covered by a single layer of electro-optic molecules (see inset transmission function). Consequently, their resonant wavelength can be shifted by the external driving voltages through the electro-optic (Pockels) effect that changes the refractive index of the modulators by Δ*n* ~ *V*_n_. When the incident beam oscillates at a given operation (OP) wavelength *λ*_op_, the transmission is modulated by the voltage (see correspondence between V, Δ*n*, and T). In an imaging experiment, the spatial and temporal control over the incident light achieves gray-scale QR codes in its far-field. In a remote sensing experiment, the ability to modulate each pixel with an unique frequency ${f}_{{{{{{{{\rm{mod}}}}}}}},{{{{{{{\rm{n}}}}}}}}}$ allows to link the RF domain with well-defined space bins. As a result, while performing a single-pixel detection of all channels at one single high-speed photodiode, a multipixel map of the environment is reconstructed by tracking, in parallel, the amplitudes of the individual transmitted modulation frequencies and their evolution over time. SLM spatial light modulator, Ch channel, QR quick response.

**Fig. 2:**
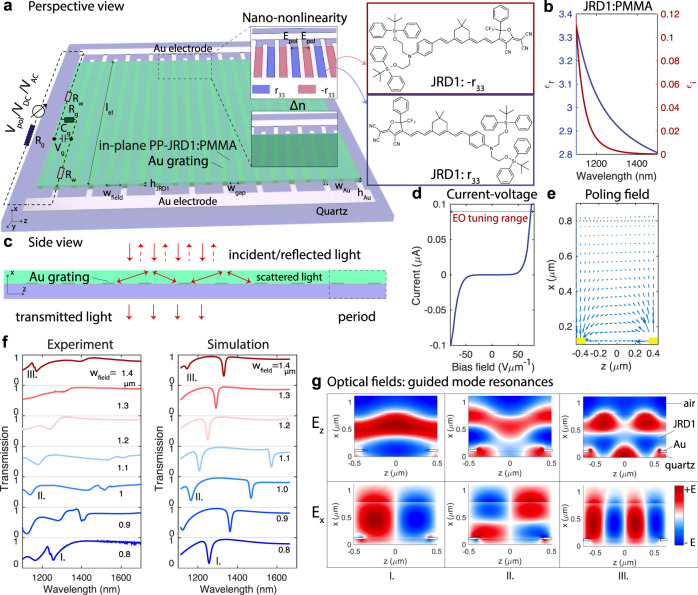
Optical and electronic properties of the resonant electro-optic modulators. **a** An array of metallic stripes with *h*_Au_ = 40–50 nm is patterned onto a quartz substrate and covered by a layer of organic electro-optic molecules JRD1 (see inset) mixed with PMMA of optical constants as shown in **b**. The electro-optic effect is accomplished by applying a poling bias to the film using the interdigitated array as described in the “Methods” section. The opposite direction of the poling field in adjacent periods is used to engineer an overall periodically poled film with alternating *r*_33_ (blue) and −*r*_33_ (red) at the nanoscale. Considering also the driving RF or DC fields, an overall homogenous refractive index change Δ*n* is achieved in all unit cells (green, see inset). The frequency-dependent electrical characteristics are modeled by a simplified equivalent circuit from *R*_w_, *R*_g_, *R*_0_, and *C*_g_. **c** The array of metallic stripes spaced by *w*_field_ = 0.8−1.4 *μ*m (displayed in different colors) sustain guided mode resonances inside the layer of JRD1:PMMA. They result from light scattered into different grating orders being guided in the slab formed by the air-JRD1:PMMA-quartz stack and subsequently reemitted. **d** Current–voltage characteristics of the junction feature bi-directional Schottky barriers formed at the metal-organic interface. The current is below 100 nA throughout the entire electro-optic tuning range up to the poling voltage of 100 V at *w*_gap_ = 1.2 *μ*m. **e** The poling field has a strong component in the *z*-direction, as shown by the field plot. The arrows indicate the direction and magnitude of the poling field. **f** The optical characteristics of the guided mode resonances are characterized experimentally and compared to the simulated results at perfectly normal incidence, which reproduce well the resonant wavelengths of the three modes. The low quality factor and depth of mode II. and III. in both measurements and simulations are attributed to losses of JRD1:PMMA (*ϵ*_**i**_ in **d**) that increase dramatically close to 1100 nm.  **g** Electro-magnetic simulations from CST Microwave studio show the real part of the x-components and z-components of the three optical modes that form within the active layer in the wavelength range from 1100 to 1700 nm, given the geometric parameters. EO electro-optic, PP-JRD1:PMMA periodically poled JRD1:PMMA, Ti titanium, Au gold, *R*_0_ external resistor, *C*_w_ resistance of one single metallic wire, *R*_g_ resistance between one single pair of metallic wires, *C*_g_ capacitance between one single pair of metallic wires.

The proposed device is depicted in Fig. 1 and is utilized to realize single-color or multicolor SLMs at high rates and achieve remote sensing by encoding space information onto the radio-frequency domain. An array of free-space electro-optic modulators controls the transmission of an incident beam in the transverse plane. Each electro-optic modulator exhibits a resonance, around which the transmission changes dramatically. Being covered by a single layer of organic electro-optic molecules, the resonant wavelength of each modulator shifts under an applied driving voltage via the *r*_33_ component of the Pockels effect (see inset for behavior around the operation wavelength *λ*_op_ as a function of voltage). A single electro-optic modulator is explained in Fig. 2a and fabricated devices are shown in Supplementary Fig. 12: metallic stripes of total thickness *h*_Au_ from titanium and gold (of thickness 15 nm and 25/35 nm, respectively and width 200 nm, see scanning electron micrograph in Supplementary Fig. 12c) are assembled into a linear array and covered by a film of organic electro-optic molecules JRD1 mixed with PMMA with 50 wt% (represented in green in Fig. 2a and shown in the photo in Supplementary Fig. 12b and the bright field image in Supplementary Fig. 12d). The film is cast post-fabrication, at which point it is not active yet. The interdigitated metallic array serves three distinct purposes: 1. it is used to render the organic thin film electro-optically active by a poling procedure during which the orientation of the organic molecules (and thereby their electro-optic coefficient *r*_33_) is oriented, at the nanoscale, along an applied DC electric field, owing to their hyperpolarizability, 2. it sustains the generation of guided mode resonances^45^ that introduce a voltage-dependent transmission of the incident beam, and 3. it is used post-activation as contacts for the RF drives of the modulators.

The organic film is poled in-plane^44^, shown in the upper inset of Fig. 2a. Adjacent unit cells of the array are poled in anti-parallel directions, yielding an in-plane periodically poled (PP) JRD1:PMMA film (in-plane PP-JRD1:PMMA) with alternating electro-optic coefficients *r*_33_ (blue) and −*r*_33_ (red). Consequently, all unit cells exhibit the same refractive index change ${{\Delta }}n(t)=-\frac{1}{2}{n}_{{{{{{{{\rm{mat}}}}}}}}}^{3}{r}_{33}{E}_{{{{{{{{\rm{AC}}}}}}}}}(t)$ (see lower inset of Fig. 2a, marked green), which depends linearly on the tuning voltage *V*_AC_ applied to the contacts ${E}_{{{{{{{{\rm{AC}}}}}}}}}(t)=\frac{{V}_{{{{{{{{\rm{AC}}}}}}}}}(t)}{{w}_{{{{{{{{\rm{gap}}}}}}}}}}$. *n*_mat_ is the material refractive index at the wavelength of the resonant mode (see Fig. 2b). A change in topography is observed after poling, see dark field and atomic force microscopy in Supplementary Fig. 12e, f. The characteristic current–voltage (I–V) curve of the modulator is shown Fig. 2d up to bias fields similar to poling fields, around of 100 V *μ*m^−1^. The device exhibits the characteristics of a metal-semiconductor-metal junction. Consequently, the tunneling current of one pixel is typically below 100 nA, even for a total modulator area of *l*_el_ × *l*_el_ = 390 *μ*m × 390 *μ*m. Details on the electronic structure of JRD1 can be found for example in Ref. ^46^. The available tuning range spans from −*E*_pol_ to +*E*_pol_. For our study, we purposefully chose large pixel arrays, as opposed to small pixels, in order to demonstrate both the low electric power consumption below 10 *μ*W (corresponding to electrical switching energies below 10 pJ at 1 *μ*s), and the ability to activate at once and drive in parallel millimeter-sized SLMs using a single JRD1:PMMA layer.

### Resonance engineering

The optical guided mode resonances (see Fig. 2c) arise through the interplay between the metallic array and the layer of JRD1:PMMA^45^ with dielectric properties as shown in Fig. 2b. The full refractive index data is provided for various concentrations of the JRD1:PMMA compound in the Supplementary Note 6 and in the data associated with this manuscript. In short, an incident beam is diffracted by the array into grating orders, which correspond, at given angles, to guided modes in the slab formed by JRD1:PMMA of *h*_JRD1_ = 690 nm and surrounded by air above and quartz below. Owing to reciprocity, the grating also scatters the guided light back which interferes with the incident light to create well-defined resonances. In both experiment and simulation, we vary the periodicity of the array *w*_field_ between 0.8 and 1.4 *μ*m and target three different guided mode resonances of different in-plane wavevectors **k**_**z**_ (I., II., and III.) in Fig. 2f which have typical field distributions with corresponding real part of their x-component and z-component as shown in Fig. 2g. Their linewidth increases with increasing thickness of the metal (see Supplementary Note. 4A). Their optical field overlaps well with the simulated DC field distribution between the two electrodes as plotted in Fig. 2e, which governs the orientation of the *r*_33_ component. We note that as a result of the spatially varying magnitude and orientation of the poling field, the electro-optic coefficient *r*_33_ and the refractive index change Δ*n* also vary spatially. A detailed discussion is provided in the Supplementary Note 3. We probe the optical properties of the modulators by shining a focused beam of light from a supercontinuum laser (SuperK Select) polarized in the *z*-direction. We record the transmission as a function of wavelength and find that our simulations reproduce well the wavelength dependence of the resonances, reported in Fig. 2f. We find that for wavelengths close to 1100 nm, the high loss of the JRD1:PMMA (see *ϵ*_**i**_ in Fig. 2b) broadens the resonances and limits their depth. We note that the broad bandwidth of the probe light (5–10 nm, shown in Supplementary Note 4D), combined with its focussing onto the sample using a lens of focal length *f* = 60 mm, obscure the real linewidth in measurements. The resonant wavelength shifts linearly with driving voltage:

1 $$\frac{{{\Delta }}{\lambda }_{{{{{{{{\rm{res}}}}}}}}}(t)}{{\lambda }_{{{{{{{{\rm{res}}}}}}}}}}=-\frac{{{\Delta }}{\omega }_{{{{{{{{\rm{res}}}}}}}}}(t)}{{\omega }_{{{{{{{{\rm{res}}}}}}}}}}=\frac{{{\Delta }}n(t)}{{n}_{{{{{{{{\rm{mat}}}}}}}}}}{{{\Gamma }}}_{c}=\frac{{{\Delta }}{n}_{{{{{{{{\rm{eff}}}}}}}}}(t)}{{n}_{{{{{{{{\rm{mat}}}}}}}}}},$$

where ${\lambda }_{{{{{{{{\rm{res}}}}}}}}}$ is the resonant frequency, Γ_c_ is the overlap factor between the optical mode, the applied DC or RF mode, and the nonlinear material. A full derivation of the non-linear interaction and the definition of the overlap factor are given in the Supplementary Note 1 and 2. We quantify the performance of our intensity modulators by their modulation strength *η*

2 $$\eta =\frac{{{\Delta }}T}{{T}_{0}}=\frac{{T}_{{{{{{{{\rm{V}}}}}}}}}-{T}_{0}}{{T}_{0}},$$

where *T*_V_ is the transmission through the modulator at given actuating voltage and *T*_0_ the transmission at 0 V. ${\eta }_{\max }$ is the maximum modulation for a given device.

### DC tuning

**Fig. 3:**
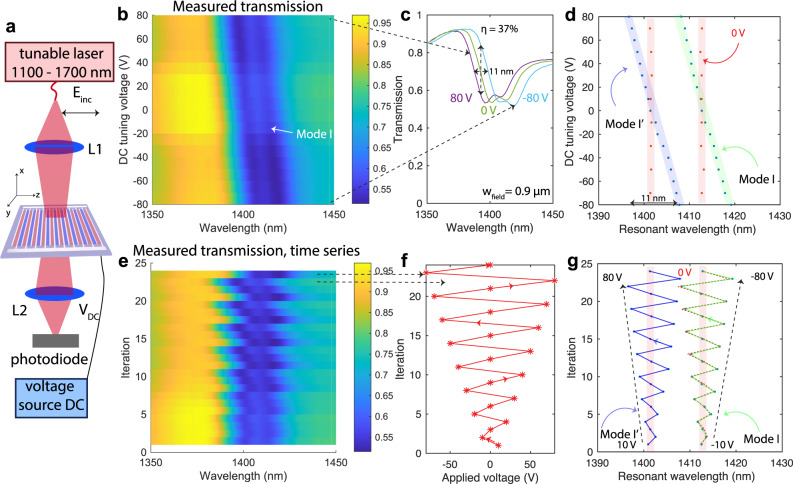
DC tuning properties of the free-space electro-optic modulators. **a** Schematic of the experimental setup used to demonstrate the tuning properties of the modulators under a DC voltage. **b** Measured transmission of a modulator with *w*_field_ = 0.9 *μ*m exhibits the expected resonance on mode I. which tunes linearly with the applied DC voltage *V*_DC_. **c** The transmission is reported for three distinct tuning voltages (−80, 0, and 80 V) from which we extract a maximal wavelength tuning of ${{\Delta }}{\lambda }_{{{{{{{{\rm{res}}}}}}}}}=11$ nm and a maximal modulation depth of ${\eta }_{\max }=37\, \%$. **d** In more detail, mode I. splits into two slightly offset modes (which we call I. and I.') due to an incident angle *θ* that slightly deviates from the normal. The resonant wavelengths attributed to mode I. (purple) and I.' (green) are extracted from **a**. A comparison with several measurements performed at 0 V (red) demonstrate a clear tuning behavior. **e**–**g** Tuning behavior of the transmission is analyzed as a time-series where in each iteration, the transmission shown in **e**, is reported when a tuning voltage as shown in **f**, is applied (the order of applied voltages is marked by the arrow). In this sequential analysis, we measure the transmission through the substrate each time after cycling the voltage between a positive and a negative value. **g** Tracking the resonant frequencies of Mode I. (purple) and I.' (green) reveal that even after applying fields as high as ±80 V, the modulator goes back to its transmission at 0 V (red) after the applied voltage is disconnected. The resonant wavelength follows the applied voltage (shown as overlay by the red dotted line). Dotted black arrows mark the direction of applied positive and negative voltages. DC direct current. Both L1 and L2 have a focal length of 60 mm.

First, we report the tuning behavior of our modulators upon an applied DC bias *V*_DC_ using the setup shown in Fig. 3a and described in the “Methods” section. We report in Fig. 3b–d the measured tuning of the resonance for mode I. at *w*_field_ = 0.9 *μ*m, which exhibits an overlap factor of Γ_c_ = 0.25. We extract a maximal tuning of the resonant frequency by ${{\Delta }}{\lambda }_{{{{{{{{\rm{res}}}}}}}}}=11$ nm over a voltage swing from +80 V to −80 V, and a maximal modulation of ${\eta }_{\max }=37\, \%$. From these results, we estimate an in-device value of *r*_33_ = 105 pmV^−1^. In more detail, mode I. splits into two slightly offset modes I. and I.’ due to an incident angle that slightly deviates from *θ* = 0°. In the Supplementary Note 4B, we show by experiment and simulations how the optical response changes with incident angle. The resonant wavelengths of these two frequencies (shown in green and purple) are represented as a function of tuning voltage in Fig. 3d, and exhibit clear tuning when compared to the resonant wavelength at *V*_DC_ = 0 V (shown in red). In Fig. 3e–g we analyse the tuning behavior of the same transmission curves as a time-series, where in each iteration the applied DC voltage is changed according to the stars in Fig. 3f in the order indicated by the arrow. When tracking the resonant wavelength of the modulator in Fig. 3g, we find that it depends linearly on the applied voltage and that even after applying fields as high as ±80 V, the modulator goes back to its transmission at 0 V after the voltage is disconnected.

### RF tuning

**Fig. 4:**
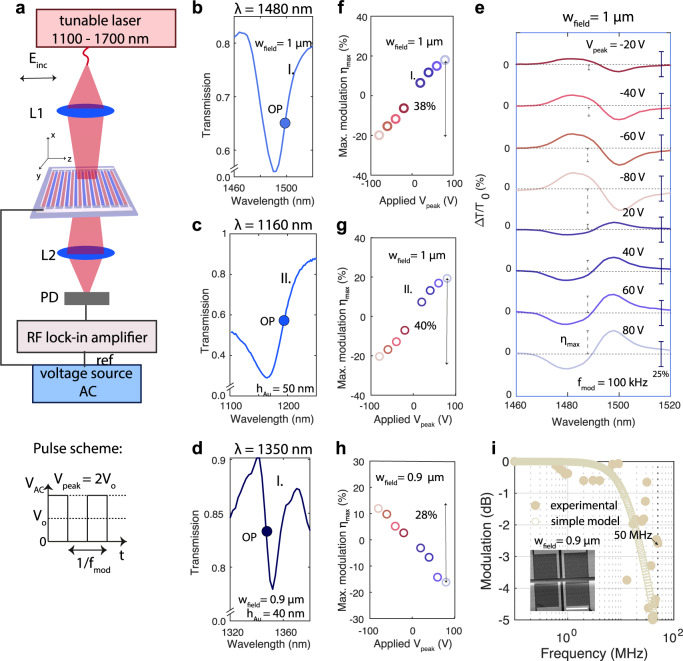
Radio-frequency tuning properties. **a** An AC voltage source drives the modulators and the introduced intensity modulation is detected with a lock-in amplifier. Inset: The utilized pulse scheme combines a DC offset voltage with an AC voltage. **b**–**d** Three different resonances are investigated: mode I. (at 1480 nm) and II. (at 1160 nm) for *w*_field_ = 1 *μ*m (*h*_Au_ = 50 nm) and mode I. (at 1350 nm) for *w*_field_ = 0.9 *μ*m (*h*_Au_ = 40 nm). The operation point (OP) is marked by the colored bullet. **e** Detailed wavelength-resolved modulation depth (solid vertical bar represents *η* = 25%) for *w*_field_ = 1 *μ*m at ${f}_{{{{{{{{\rm{mod}}}}}}}}}=100$ kHz reveals a linear dependence of the maximum intensity modulation ${\eta }_{\max }$ (dashed vertical bars) on the applied *V*_peak_ (shown in **f**, for mode I. and **g** for mode II.)). A similar behavior is reported for a second modulator with *w*_field_ = 0.9 *μ*m (shown in **h**). In all devices, we achieve a maximal modulation strength up to 30–40%. **i** The modulation strength (normalized to its value at 100 kHz) is reported for one pixel of the 2 × 2 SLM shown in the inset (scale bar = 200 *μ*m) as a function of ${f}_{{{{{{{{\rm{mod}}}}}}}}}$, where the full (empty) circles are experimental (analytical) data. A 3 dB decay close to 50 MHz is observed (further details in Supplementary Note 4F). RF radio frequency, AC alternating current.

Now we discuss the performance of our devices at radio frequencies ${f}_{{{{{{{{\rm{mod}}}}}}}}}$, up to 50 MHz. We drive the modulators with an RF source (pulser Agilent 8114A, maximal frequency 50 MHz) and demodulate the transmitted intensity at the modulation frequency of the pulses, as shown in Fig. 4. We demonstrate the broadband character of the Pockels effect in the organic material exemplarily at three chosen resonances, dictated primarily by the periodicity of the interdigitated array, that span from 1100 to 1500 nm, shown in Fig. 4b (at 1480 nm, mode I. for *w*_field_ = 1 *μ*m), *c* (at 1160 nm, mode II. for *w*_field_ = 1 *μ*m, Γ_*c*_ = 0.3) and d (at 1350 nm, mode II. for *w*_field_ = 0.9 *μ*m, Γ_c_ = 0.25). The pulse scheme consists of an overall DC voltage offset *V*_o_, and an additional RF voltage with *V*_peak_ = 2*V*_o_, as shown in the inset of Fig. 4a. In Fig. 4e we report an exemplary wavelength-resolved modulation curve for *V*_peak_ = [−80 V, 80 V] at a modulation frequency ${f}_{{{{{{{{\rm{mod}}}}}}}}}=100$ kHz, while operating around the resonances shown in Fig. 4b. We observe a low-noise modulation (its maximum value is marked with the vertical dashed marker), which switches sign around the resonant wavelength and is strongest where the slope in the transmission curve is highest, as expected. Moreover, it flips sign when switching the polarity of the applied modulation voltage, as expected from the exploited Pockels effect, which is linear in field amplitude. From the modulation data, we plot the maximal modulation strength ${\eta }_{\max }$ for *w*_field_ = 1 *μ*m in Fig. 4f, g, and for *w*_field_ = 0.9 *μ*m in Fig. 4h, respectively. For the resonances at 1480 nm, 1350 nm and 1160 nm we report a maximum modulation of ${\eta }_{\max }=38 \%$, 28% and 40%, respectively. We demonstrate experimentally that a 2 × 2 SLM device can be operated up to a frequency of ${f}_{{{{{{{{\rm{mod}}}}}}}}}=50$ MHz where a 3-dB decay of the modulation is observed, marked by the full circles in Fig. 4i. In the Supplementary Note 4F, we provide extensive optical and electronic measurements that show how a series resistance influences the cut-off frequency of the pulser-cable-sample system. Additionally, we derive a simple low-frequency equivalent circuit model loaded with an external resistor for the pulser-cable-sample system, marked by empty circles in Fig. 4i. We note that for operation at frequencies beyond the ones discussed here, this model is inappropriate and a high-frequency transmission line model that accounts also for the cable line inductance is necessary. Finally, we experimentally quantify thermo-optic tuning effects and show that their contribution is negligible as compared to the electro-optic effect in the Supplementary Note 4C.

### Single-color and multicolor SLMs

**Fig. 5:**
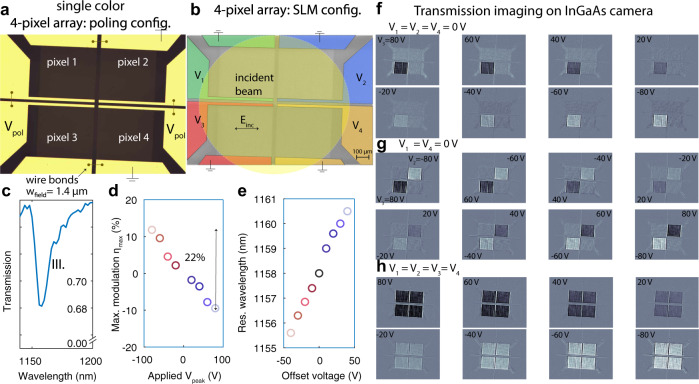
Imaging properties of the monolithic 4-pixel spatial light modulator. **a** Optical microscope image describes the geometrical and electrical configuration of the 4-pixel array (yellow regions are the bonding pads that connect the metallic grating). All four pixels are identical and connected in parallel during the poling procedure and are thereby all activated in one single run. **b** False-color scanning electron micrograph shows the color-coded control voltages *V*_1_ − *V*_4_ and the incident beam that probes the entire SLM in transmission. **c** For this demonstration, we choose to operate on mode III. of a modulator with *w*_field_ = 1.4 *μ*m, which exhibits a maximal modulation strength of 22% at 100 kHz shown in **d**, and a tuning of the resonant wavelength by 5 nm, shown in **e**. **f**–**h** Pixels are individually addressed by the set of voltages *V*_1_, *V*_2_, *V*_3_, *V*_4_ and the intensity distribution of an incident beam after transmission through the SLM is recorded with a InGaAs camera at low speed (25 Hz, limited by the camera). The transmission experiments confirm a successful concomitant poling of all four pixels (thereby of an area 780 × 780 *μ*m^2^), comparable performance of all pixels, as well as independent control over their transmission properties. All pictures are generated by subtracting the transmitted intensity pattern when the modulator is not biased i.e., *V*_1_ = *V*_2_ = *V*_3_ = *V*_4_ = 0. SLM spatial light modulator, InGaAs indium gallium arsenide.

In the following we combine several modulators into arrays: in a first example discussed here four of equal periodicity (*w*_field_ = 1.4 *μ*m) into a 4-pixel array, which features a total active area of 780 × 780 *μ*m^2^. All pixels are poled in parallel at once in one single run, using the voltage configuration of Fig. 5a. For remote control, each pixel is instead contacted individually (voltages *V*_1_ to *V*_4_), as shown in Fig. 5b. A large area incident beam has a central emission wavelength of *λ* = 1154 nm, and operates at the high-sensitivity working point marked in Fig. 5c, with a maximum ${\eta }_{\max }=22 \%$ and a resonant wavelength tuning of ${{\Delta }}{\lambda }_{{{{{{{{\rm{res}}}}}}}}}=5$ nm over a swing of −40 V to 40 V. The modulated beam is imaged using an indium gallium arsenide (InGaAs) camera at 25 frames per second, limited by the camera. In Fig. 5f–h, we report multiple intensity profiles under different combinations of the actuating voltages *V*_1_ to *V*_4_ and at eight representative voltage levels, which result into well discernible transmission patterns through the SLM. The depicted intensity profiles have been post-processed by subtracting the transmission through the modulator at voltages *V*_1_ = *V*_2_ = *V*_3_ = *V*_4_ = 0. From one single pixel in Fig. 5f, we augment to two pixels in Fig. 5g and then four pixels concomitantly actuated pixels in Fig. 5h. The individual pixels exhibit comparable intensity strengths at a fixed voltage, which confirms their homogenous poling, and the straight-forward ability to pole a millimeter area with good uniformity. In the Supplementary Note 4B, we show by experiment and simulations how the optical response changes with incident angle and discuss how this influences the angle of acceptance of the electro-optic modulators, which we find to be *ϕ* = ±10° and *θ* = ±1° (see Supplementary Figs. 3 and 4). In a second example shown in the Supplementary Note 5, five pixels of distinct periodicities are assembled into a multi-color SLM. The Supplementary Movie 1 associated with this manuscript demonstrates its operation. It consists of a stack of images that represent the raw intensity modulation measured with the camera, while tuning the laser in resonance with the individual pixels of the multicolor SLM.

### Remote sensing by single-pixel imaging

**Fig. 6:**
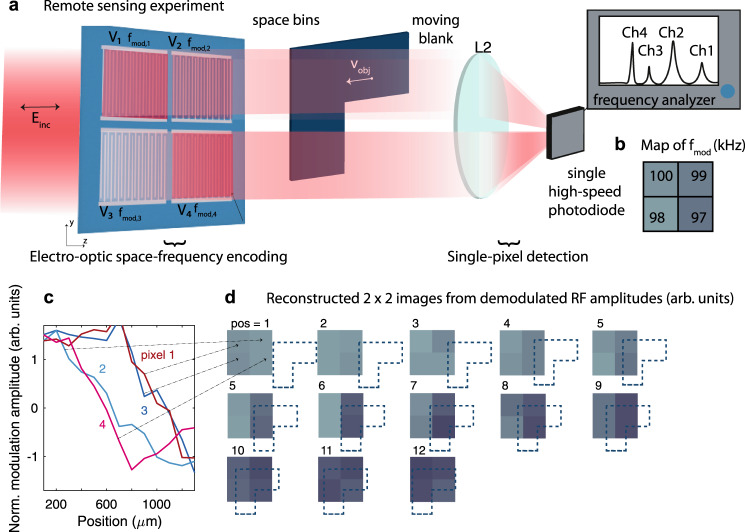
Multipixel remote sensing by single-pixel detection: encoding space bins onto distinct RF frequencies. **a** An incident beam is divided into four space bins by the four pixels of the SLM and the light passing through each pixel is modulated with a distinct *f*_mod,n_, as shown in **b** Thereby, a one-to-one correspondence is created between each space bin and a radio-frequency. An intensity measurement of all four channels at one single high-speed detector is demodulated at the individual modulation frequencies to reconstruct the transmission through the environment. In a proof-of-concept experiment, an L-shaped blank is traversing the *x*-*z*-plane between the SLM and the single-pixel detector. **c** Demodulated RF amplitudes are reported as a function of the position of the blank. **d** These amplitudes are then further bundled to create two-dimensional pictures of the transmitted light. The dotted lines represent the position of the blank with respect to the four space bins, and the intensity of the pixels represent the amplitudes of the demodulated RF fields in arbitrary units.

In a second experiment, we use our SLM for remote sensing on parallel channels. The core concept is that the SLM divides a three-dimensional space into *n* distinct space bins (here *n* = 4, the number of pixels), as shown in Fig. 6a. By modulating the four pixels at individual frequencies (${f}_{{{{{{{{\rm{mod}}}}}}}},1}=100\,$ kHz, ${f}_{{{{{{{{\rm{mod}}}}}}}},2}=99\,$ kHz, ${f}_{{{{{{{{\rm{mod}}}}}}}},3}=98\,$ kHz, ${f}_{{{{{{{{\rm{mod}}}}}}}},4}=97\,$ kHz), a one-to-one correspondence is established between the distinct space bins and their associated radio frequency (Fig. 6b). The beam then traverses a space that is monitored and is subsequently detected at a single pixel high-speed InGaAs photodiode (the collection lens has a focal length of 60 mm). As a result of the transfer of information from the spatial to the RF domain, a deconvolution of the individual modulation frequencies and their amplitudes permits to reconstruct a time-resolved or position-resolved image of the transverse plane without the need of a camera^28^. When a L-shape blank is traversing the modulated beam in the transversal direction, it introduces changes in the intensity of the transmitted light and thereby also of the radio frequencies. The demodulated amplitudes (here measured subsequently, due to the unavailability of four distinct RF sources) are shown in Fig. 6c. In Fig. 6d we show the reconstructed two-dimensional maps of the transmitted beam. The position of the blank is marked by the dotted line and clearly influences the transmission of the individual pixels. Our preliminary demonstration underlines the key characteristics of this measurement scheme, discussed in the “Methods” section.

In conclusion, we have demonstrated a 2 × 2-pixel single-color SLM and a 1 × 5 pixel multicolor SLM, featuring an acceptance angle of *ϕ* = ±10° and *θ* = ±1°, which exploits broadband high-performance electro-optic molecules as an active medium to impart an intensity modulation up to 40% to an incident beam at speeds as high as 50 MHz. We deploy the spatial light modulator of total area of 780 × 780 *μ*m^2^ in two real-life scenarios, for image projection and to remotely probe the environment on parallel channels. The unique properties of the organic film, including the ability to engineer its nonlinearity at will, at the nanoscale, its compatibility with broad resonances (and thereby broadband lasers), and its high-speed could open long-anticipated applications that benefit from—or require—straight-forward up-scaling, simple fabrication and low power consumption: imaging, virtual reality, laser ranging, and sensing. While further studies are needed to push the modulation speed of free-space modulators towards the microwaves and terahertz, the hybrid organic electro-optic integration with multipixel nanophotonic technology demonstrated here already shows a path to create more efficient, faster and more customized SLMs. Finally, brand-new organic electro-optic materials HLD1:HLD2 have been reported recently to have excellent temperature and chemical resilience in long-term aging tests^39^, thereby overcoming any remaining issues for organic electro-optic materials to be deployed into a much greater variety of instrumentation at large scales.

## Methods

### Sample fabrication and electric field poling of the devices

The free-space modulators were fabricated on 1 mm thick quartz substrates by electron beam lithography (ZEP520A) and subsequent electron beam evaporation of a metallic wires of thickness 15 nm titanium and 25/35 nm gold and width *w*_Au_ = 200 nm. After lift-off, the samples were coated by the JRD1:PMMA mixture which had been prepared beforehand. A 50%wt mixture of JRD1 powder and PMMA in solid form was first mixed with 1,1,2-trichlorethane (5% wt) for 8 h in a swirling machine. Afterwards, the solution was filtered using 0.2 *μ*m PTFE-filters before being deposited on-chip by spin-coating at 1000 rpm. After coating, the film was dried in a vacuum oven at 85 °C for 24 h. After drying, a semitransparent, clean and homogenous film of 690 nm thickness was measured on the final samples. The electric field poling of the organic film was then performed inside a DC probe station under vacuum. All samples were first characterized regarding their typical IV-curves, which all exhibited the typical diode-like characteristics shown in the main text. A poling field of typically 100 V *μ*m^−1^ (below the breakdown field of the material) was applied to the interdigitated electrodes, which were heated above glass temperature 97 °C. The poling field was kept for typically 10 min at poling temperature, after which the samples were cooled down to room temperature. Finally, poled samples were inspected both in bright and dark field microscopy, as well as using atomic force microscopy. As shown, the film changes conformation quite significantly, which can be used as an indication of efficient poling. In the case of the multipixel SLMs, all pixels are contacted in parallel prior to electric field poling by wire bonding. Consequently, during the poling procedure, all pixels of one single SLM experience the same poling field and the nonlinearity in all pixels is established in one single run. Given this activation procedure, it is essential that all pixels feature comparable internal resistances.

### Experimental setup

A DC/AC voltage is applied to one contact of the modulator, while the other contact is grounded. A slow (DET20C2) or fast (FPD310-FS-NIR) photodiode monitors the transmitted power. For the RF measurements, we use a RF pulsed source to drive the modulators up to 50 MHz and a Zurich Instruments RF lock-in amplifier (MFLI, 5 MHz and UHFLI, 600 MHz) to demodulate the transmitted intensity at the modulation frequency of the pulses. We use a tunable laser (super-K Select) to probe the modulation characteristics of our modulators. Its linewidth is described in detail in Fig. 4 of the Supplementary Information, and exceeds 4 nm and can range up to 11.5 nm. The ability of our modulators to introduce significant intensity modulation even to a broadband incident beam are an experimental proof that the modulators are—in principle—compatible with compact laser diodes, which have relatively broad linewidths. Even more, they can accommodate wavelength tolerances in the fabrication of laser diodes which are typically up to 10 nm (e.g., Eblana Photonics EP1310-ADF-B laser diode). This property outperforms typical demonstrations of electro-optic free-space transducers which trade a very specific and narrow operation point for high intensity/phase tuning properties.

### Relationship between channels and integration time in remote sensing

The frequency resolution of the individual channels is given by $\delta f=\frac{1}{{t}_{{{{{{{{\rm{int}}}}}}}}}}$, where *t*_int_ is the total integration time per pixel. A resolution around 0.1 kHz requires 10 ms integration time and is sufficient to resolve modulation frequencies spaced by 1 kHz. In our case, the demonstrated modulation bandwidth up to 50 MHz would accommodate—in principle—the modulation frequencies of 50,000 pixels. However, having the ability to modulate the individual pixels at speeds up to the terahertz not only allows to increase the number of pixels but also to reduce the integration time per pixel, by spacing them further apart in the RF domain.

## Supplementary information

Supplementary Information

Description of Additional Supplementary Files

Supplementary Movie 1

## Data Availability

The main dataset contained within this paper, as well as the material parameters of JRD1:PMMA depicted in Supplementary Fig S10 and 11 are available in the Zenodo database at 10.5281/zenodo.5140234.

## Code availability

The code used to plot the data within this paper is available in the Zenodo database at 10.5281/zenodo.5140234.
