# Supplementary material for: Electro-optic spatial light modulator from an engineered organic layer
Source: Nat Commun. 2021 Oct 11;12:5928. doi: 10.1038/s41467-021-26035-y (PMC8505481; doi:10.1038/s41467-021-26035-y)
Supplement: Supplementary file 1 — Supplementary Information [file 41467_2021_26035_MOESM1_ESM.pdf]

Supplementary material for: Electro-optic spatial light modulator from an engineered organic layer

Ileana-Cristina Benea-Chelmus*

Harvard John A. Paulson School of Engineering and Applied Sciences, Harvard University, Cambridge, MA, USA

Maryna L. Meretska

Harvard John A. Paulson School of Engineering and Applied Sciences, Harvard University, Cambridge, MA, USA

Delwin L. Elder

University of Washington, Department of Chemistry, Seattle, WA, USA

Michele Tamagnone

Harvard John A. Paulson School of Engineering and Applied Sciences, Harvard University, Cambridge, MA, USA

Larry R. Dalton

University of Washington, Department of Chemistry, Seattle, WA, USA

Federico Capasso†

Harvard John A. Paulson School of Engineering and Applied Sciences, Harvard University, Cambridge, MA, USA

(Dated: September 6, 2021)

Abstract

Tailored nanostructures provide at-will control over the properties of light, with applications in imaging and spectroscopy. Active photonics can further open new avenues in remote monitoring, virtual or augmented reality and time-resolved sensing. Nanomaterials with $\chi^{(2)}$ nonlinearities achieve highest switching speeds. Current demonstrations typically require a trade-off: they either rely on traditional $\chi^{(2)}$ materials, which have low non-linearities, or on application-specific quantum well heterostructures that exhibit a high $\chi^{(2)}$ in a narrow band. Here, we show that a thin film of organic electro-optic molecules JRD1 in polymethylmethacrylate combines desired merits for active free-space optics: broadband record-high nonlinearity (10-100 times higher than traditional materials at wavelengths 1100-1600 nm), a custom-tailored nonlinear tensor at the nanoscale, and engineered optical and electronic responses. We demonstrate a tuning of optical resonances by $\Delta\lambda = 11$ nm at DC voltages and a modulation of the transmitted intensity up to 40%, at speeds up to 50 MHz. We realize 2×2 single- and 1×5 multi-color spatial light modulators (SLMs). We demonstrate their potential for imaging and remote sensing. The compatibility with compact laser diodes, the achieved millimeter size and the low power consumption are further key features for laser ranging or reconfigurable optics.

[*] cristinabenea@g.harvard.edu

[†] capasso@seas.harvard.edu

SUPPLEMENTARY NOTE 1. SECOND-ORDER NONLINEAR OPTICAL INTERACTION: ELECTRO-OPTIC COUPLING

A radio-frequency tuning field $E_{\mathrm{rf}}(t)$ is used in this work to introduce a high-speed modulation of the resonant frequency of an array of nanoresonators via its interaction with a resonant optical field that oscillates in the near-infrared $E_{\mathrm{opt}}(t)$. Here, we derive from first principles the expected frequency shift, which we will show to depend on the electro-optic coefficient of the material, the distribution of the non-linearity on-chip upon electric field poling, as well as the overlap between the two interacting modes. We follow closely the methodology provided in reference [1], but we choose to lay it out here in detail for completeness.

In our chip design, we exploit the $\chi^{(2)}_{33}$ component of the nonlinear susceptibility tensor. In our derivation, we neglect all other components, such as $\chi^{(2)}_{13}$, which is known to be non-zero, but has a much lower magnitude. For now, we focus to derive the main physics. We will treat device-specific characteristics separately, such as local variations of $\chi^{(2)}_{33}$. We will show that the generated non-linear polarisation $P^{(2)}$ is the origin of a net shift of the resonant frequency of $E_{\mathrm{opt}}(t)$.

The total field in the near-field of the resonators is $E(t)$. The nonlinear interaction energy arises from the nonlinear polarisation $P^{(2)}(t) = \chi^{(2)}_{33} E(t)^2$:

$$\langle U^{(2)}(t)\rangle = \int dV \int dt \langle E(t) \frac{\partial P^{(2)}(t)}{\partial t}\rangle \tag{1}$$

and $\langle\rangle$ is the average energy over time. We further derive the equations of motion by passing into the quantum picture, where new fields are created from the vacuum as a result of the interaction. The corresponding interaction Hamiltonian can be derived from the expression of the energy by using the electric field operators in the second quantisation.

In our scenario, a resonant optical mode interacts with a well-localized radio-frequency field that is applied to the structure via interdigitated electrodes. We describe the optical mode as a scalar classical, coherent tone $E_{\mathrm{opt}}(t) = \sqrt{\frac{\hbar\omega}{2\epsilon_{\mathrm{opt}}V_{\mathrm{opt}}}}(u_{\mathrm{opt}}(x,y,z)\alpha(\omega)e^{-i\omega t} + h.c.)$. Simmilarly, we describe the tuning field as $E_{\mathrm{rf}}(t) = \sqrt{\frac{\hbar\omega_{\mathrm{rf}}}{2\epsilon_{\mathrm{rf}}V_{\mathrm{rf}}}}(u_{\mathrm{rf}}(x,y,z)\alpha(\omega_{\mathrm{rf}})e^{-i\omega_{\mathrm{rf}} t} + h.c.)$. To ease the notation, we omit to write the explicit dependency of all fields also on x, y, z. $u_{\mathrm{opt}}(x,y,z)$ and $u_{\mathrm{rf}}(x,y,z)$ are the three-dimensional spatial field distributions that obey the normalisation $\int dV|u_{\mathrm{rf}}(x,y,z)|^2 = V_{\mathrm{rf}}$, $\int dV|u_{\mathrm{opt}}(x,y,z)|^2 = V_{\mathrm{opt}}$, ϵ_{opt} and ϵ_{rf} are the

permittivities, V_{opt} and V_{rf} are the effective mode volumes and $\alpha(\omega)$ and $\alpha(\omega_{\text{rf}})$ are the coherent amplitudes at the two frequencies. We assume without loss of generality that $u_{\text{opt}}(x, y, z)$, $u_{\text{rf}}(x, y, z)$, $\alpha(\omega_{\text{opt}})$ and $\alpha(\omega_{\text{rf}})$ are real-valued.

A result of the interaction is that side-bands at frequencies $\omega + \omega_{\text{rf}}$ and $\omega - \omega_{\text{rf}}$ will be generated from vacuum through sum and difference frequency generation. We describe these initial vacuum modes as quantum fields that have an amplitude and a spatial distribution that are well approximated by vacuum field of the NIR mode $E_{vac}^{opt} = \sqrt{\frac{\hbar\omega}{2\epsilon_{\text{opt}} V_{\text{opt}}}}$. Consequently, we assume for the blue-detuned sideband $\hat{E}_{\text{opt+rf}}(t) = \sqrt{\frac{\hbar\omega}{2\epsilon_p V_p}}(u_{\text{opt}}(x, y, z)\hat{a}(\omega + \omega_{\text{rf}})e^{-i(\omega+\omega_{\text{rf}})t} + h.c.)$ and for the red detuned we have $\hat{E}_{\text{opt-rf}}(t) = \sqrt{\frac{\hbar\omega}{2\epsilon_p V_p}}(u_{\text{opt}}(x, y, z)\hat{a}(\omega - \omega_{\text{rf}})e^{-i(\omega-\omega_{\text{rf}})t} + h.c.)$. $\hat{a}(\omega)$ ($\hat{a}(\omega)^\dagger$) are the annihilation (creation) operators. The total field is therefore $\hat{E}(t) = E_{\text{opt}}(t) + E_{\text{rf}}(t) + \hat{E}_{\text{opt+rf}}(t) + \hat{E}_{\text{opt-rf}}(t)$, and $P^{(2)}(t) = \chi_{33}^{(2)}(E_{\text{opt}}(t) + E_{\text{rf}}(t) + \hat{E}_{\text{opt+rf}}(t) + \hat{E}_{\text{opt-rf}}(t))^2$. We find:

$$\hat{H}_{\text{I}} = \int dV \frac{2}{3}\chi_{33}^{(2)}(E_{\text{opt}}(t) + E_{\text{rf}}(t) + \hat{E}_{\text{opt+rf}}(t) + \hat{E}_{\text{opt-rf}}(t))^3. \tag{2}$$

Since we use the radio-frequency wave as a linear control field, we consider only the terms which are linear with $E_{\text{rf}}(t)$, and neglect counter-rotating terms. This corresponds to the time-averaging in the classical picture. The final interaction Hamiltonian is:

$$\hat{H}_{\text{I}} = 2\epsilon_0\chi_{33}^{(2)}\sqrt{\frac{\hbar\omega_{\text{opt}}^2\omega_{\text{rf}}}{8\epsilon_{\text{opt}}^2\epsilon_{\text{rf}}V_{\text{opt}}^2V_{\text{rf}}}}\alpha(\omega)\alpha(\omega_{\text{rf}})\int dV|u_{\text{opt}}(x, y, z)|^2u_{\text{rf}}(x, y, z)(\hat{a}(\omega + \omega_{\text{rf}}) + \hat{a}(\omega - \omega_{\text{rf}}) + h.c. \tag{3}$$

We define the single photon electro-optic coupling rate $g_{\text{eo}}(\omega)$ as:

$$g_{\text{eo}}(\omega) = 2\epsilon_0\chi_{33}^{(2)}\sqrt{\frac{\hbar\omega^2\omega_{\text{rf}}}{8\epsilon_{\text{opt}}^2\epsilon_{\text{rf}}V_{\text{opt}}^2V_{\text{rf}}}}\int dV|u_{\text{opt}}(x, y, z)|^2u_{\text{rf}}(x, y, z) \tag{4}$$

with the integral that describes the effective overlap volume $V_{\text{overlap}} = \int dV|u_{\text{opt}}(x, y, z)|^2u_{\text{rf}}(x, y, z)$. Using $\chi_{33}^{(2)} = -\frac{1}{2}n_{\text{mat}}^4 r_{33}$ (n_{mat} is the material index at the probe wavelength, r_{33} the electro-optic coefficient), and Γ_c the overlap between the two interacting fields, we simplify

$$g_{\text{eo}}(\omega) = \frac{1}{2}n_{\text{mat}}^2 r_{33}\omega\sqrt{\frac{\hbar\omega_{\text{rf}}}{2\epsilon_{\text{rf}}V_{\text{rf}}}}\Gamma_c \tag{5}$$

Finally, we define the multi-photon electro-optic coupling rate as

$$g_{\text{eo}}^{\text{tot}}(\omega) = \alpha(\omega)\alpha(\omega_{\text{rf}})g_{\text{eo}}(\omega). \tag{6}$$

Therefore,

$$\hat{H}_{\mathrm{I}} = \hbar g_{\mathrm{eo}}^{\mathrm{tot}}(\omega)(\hat{a}(\omega+\omega_{\mathrm{rf}}) + \hat{a}(\omega-\omega_{\mathrm{rf}}) + h.c.). \tag{7}$$

As shown in Fig. 2 e and 2 g of the main text, both the radio-frequency field as well as the near-infrared optical field are well confined to the near-field region of the resonators. Due to translation symmetry of the modes along the y-plane we can factorize $u_{\mathrm{opt}}(x,y,z) = v_{\mathrm{opt}}(x,z)w_{\mathrm{opt}}(y)$ and $u_{\mathrm{rf}}(x,y,z) = v_{\mathrm{rf}}(x,z)w_{\mathrm{rf}}(y)$, with $w_{\mathrm{opt}}(y) = w_{\mathrm{rf}}(y) = 1$ everywhere in the gap. Consequently, the overlap Γ_c can be approximated by the two-dimensional overlap of the mode profiles in the transverse xz-plane:

$$\Gamma_c = \frac{\int dV |u_{\mathrm{opt}}(x,y,z)|^2 u_{\mathrm{rf}}(x,y,z)}{\int dV |u_{\mathrm{opt}}(x,y,z)|^2} = \frac{\int dA |v_{\mathrm{opt}}(x,z)|^2 v_{\mathrm{rf}}(x,z) \int dy |w_{\mathrm{opt}}(y)|^2}{\int dA |v_{\mathrm{opt}}(x,z)|^2 \int dy |w_{\mathrm{opt}}(y)|^2} = \frac{A_{\mathrm{overlap}}}{A_{\mathrm{opt}}} \tag{8}$$

where A_{overlap} is the effective overlap area and A_{opt} is the effective mode area of the optical mode in the transverse plane.

SUPPLEMENTARY NOTE 2. ELECTRO-OPTICALLY INDUCED RESONANCE SHIFT

We use the interaction Hamiltonian above to analyse the amplification of the side bands from the vacuum. For this purpose we start from the equations of motion in the Heisenberg picture:

$$\frac{d}{dt}\hat{a}(\omega+\omega_{\mathrm{rf}}) = \frac{i}{\hbar}[\hat{H}_{\mathrm{I}}, \hat{a}(\omega+\omega_{\mathrm{rf}})] \tag{9}$$

$$\frac{d}{dt}\hat{a}(\omega-\omega_{\mathrm{rf}}) = \frac{i}{\hbar}[\hat{H}_{\mathrm{I}}, \hat{a}(\omega-\omega_{\mathrm{rf}})] \tag{10}$$

Then

$$\frac{d}{dt}\hat{a}(\omega+\omega_{\mathrm{rf}}) = i g_{\mathrm{eo}}^{\mathrm{tot}}(\omega) \tag{11}$$

$$\frac{d}{dt}\hat{a}(\omega-\omega_{\mathrm{rf}}) = i g_{\mathrm{eo}}^{\mathrm{tot}}(\omega). \tag{12}$$

While the interaction time t_{int} is equal to the propagation time of the optical field through the modulator $t_{\mathrm{int}} = \frac{h_{\mathrm{JRD1}} n_{\mathrm{g}}}{c_0}$, with n_{g} the group index of the optical mode and h_{JRD1} the

height of the non-linear material. We solve the above differential equations and find that the side-bands have the following amplitudes after the interaction:

$$\hat{a}(\omega + \omega_{\rm rf}, t_{\rm int}) = i g_{\rm eo}^{\rm tot}(\omega) t_{\rm int} \tag{13}$$

$$\hat{a}(\omega - \omega_{\rm rf}, t_{\rm int}) = i g_{\rm eo}^{\rm tot}(\omega) t_{\rm int} \tag{14}$$

Therefore, after interaction, the total resulting electric field is:

$$\hat{E}_{\rm tot}(t) = E_{\rm opt}(t) + \hat{E}^{(2)}_{\rm opt}(t) \tag{15}$$

and

$$E^{(2)}_{\rm opt}(t) = i\sqrt{\frac{\hbar\omega}{2\epsilon_{\rm opt}V_{\rm opt}}} u_{\rm opt}(x,y,z) g_{\rm eo}^{\rm tot}(\omega) t_{\rm int}(e^{-i\omega t} + h.c.)(e^{-i\omega_{\rm rf} t} + h.c.) \tag{16}$$

We define the phase delay: $\Delta\phi(t) = \frac{1}{2} n_{\rm mat}^2 r_{33}\omega\Gamma_c t_{\rm int} E_{\rm rf}(t)$, with $E_{\rm rf}(t) = E_{\rm rf}(e^{-i\omega_{\rm rf}t} + h.c.)$ and approximate $i\Delta\phi(t) = e^{i\Delta\phi(t)} - 1$ and find that

$$E_{\rm tot}(t) = \sqrt{\frac{\hbar\omega}{2\epsilon_{\rm opt}V_{\rm opt}}} u_{\rm opt}(x,y,z)\alpha(\omega)(e^{-i(\omega t - \Delta\phi(t))} - h.c.) \tag{17}$$

From this equation, we indeed retrieve that the interaction introduces a phase delay onto the optical beam. This phase delay is the origin of the frequency shift experienced by the nanoresonators. Consequently, we find that the high-speed tuning of the resonant frequency obeys the following relation:

$$\Delta\omega_{\rm res}(t) t_{\rm int} = \Delta\phi(t) \tag{18}$$

$$\frac{\Delta\omega_{\rm res}(t)}{\omega_{\rm res}} = \frac{1}{2} n_{\rm mat}^2 r_{33}\Gamma_c E_{\rm rf}(t). \tag{19}$$

We use further the common convention that $\Delta n(t) = -\frac{1}{2} n_{\rm mat}^3 r_{33} E_{\rm rf}(t)$ and conclude

$$\frac{\Delta\lambda_{\rm res}}{\lambda_{\rm res}} = -\frac{\Delta\omega_{\rm res}(t)}{\omega_{\rm res}} = \frac{\Delta n(t)}{n_{\rm mat}}\Gamma_c. \tag{20}$$

SUPPLEMENTARY NOTE 3. LOCAL SECOND ORDER NONLINEAR SUSCEPTIBILITY $\chi_{33}^{(2)}(x,z)$

The nonlinearity of the material is established in our devices via electric field poling. As shown in Fig. 2 of the main text, the poling field lines are not uniformly parallel to the z-axis everywhere in the non-linear material. As a result, both the local orientation and the magnitude of $\chi_{33}^{(2)}(x,z)$ depend on the location in the xz-plane (the local "z-plane" of the crystal can be thought of as changing locally, hence $\chi_{33}^{(2)}$ is not always parallel to the geometrical z-axis). However, since the molecules arrange along this axis, we choose to still call it $\chi_{33}^{(2)}$, even though - technically - it is not parallel to the z-axis. Consequently, we now describe the second order susceptibility as a vector field that is aligned with the electric field lines described by a unit vector $\vec{e}_{\mathrm{DC}}(x,z)$ and has a magnitude described by $v_{\mathrm{DC}}(x,z)$ which is linearly proportional to the local amplitude of the poling field $\vec{\chi}_{33}^{(2)}(x,z) = \chi_{33}^{(2)}(x,z)\vec{e}_{\mathrm{DC}}(x,z) = \chi_{33}^{(2)}v_{\mathrm{DC}}(x,z)\vec{e}_{\mathrm{DC}}(x,z)$, with $|\vec{e}_{\mathrm{DC}}(x,z)| = 1$. This linear dependence on the poling field has been demonstrated experimentally, e.g. here [2, 3]. In addition, the interacting fields also have both x- and z-components. Consequently, we describe also the interacting fields as vector fields with a local polarisation parallel to $\vec{E}_{\mathrm{opt}}(x,z)$ and $\vec{E}_{\mathrm{rf}}(x,z)$ ($|\vec{e}_{\mathrm{opt}}(x,z)| = 1$ and $|\vec{e}_{\mathrm{rf}}(x,z)| = 1$) and introduce the in-plane dependence explicitly:

$$\vec{E}_{\mathrm{opt}}(x,z,t) = \sqrt{\frac{\hbar\omega}{2\epsilon_{\mathrm{opt}}V_{\mathrm{opt}}}}(v_{\mathrm{opt}}(x,z)\alpha(\omega)e^{-i\omega t} + h.c.)\vec{E}_{\mathrm{opt}}(x,z) \tag{21}$$

$$\vec{E}_{\mathrm{rf}}(x,z,t) = \sqrt{\frac{\hbar\omega_{\mathrm{rf}}}{2\epsilon_{\mathrm{rf}}V_{\mathrm{rf}}}}(v_{\mathrm{rf}}(x,z)\alpha(\omega_{\mathrm{rf}})e^{-i\omega_{\mathrm{rf}}t} + h.c.)\vec{E}_{\mathrm{rf}}(x,z). \tag{22}$$

Finally, the interaction will occur between the field components that are parallel with the nonlinearity established by poling and hence collinear with $\vec{e}_{\mathrm{DC}}$. For the radio frequencies we investigate here, we assume that $\vec{E}_{\mathrm{rf}} \parallel \vec{e}_{\mathrm{DC}}$ and $v_{\mathrm{rf}}(x,z) = v_{\mathrm{DC}}(x,z)$, since $\lambda_{\mathrm{rf}} >> w_{gap}$.

With $\hat{\vec{E}}_{\mathrm{tot}}(x,z,t) = \vec{E}_{\mathrm{opt}}(x,z,t) + \vec{E}_{\mathrm{rf}}(x,z,t) + \hat{\vec{E}}_{\mathrm{opt+rf}}(x,z,t) + \hat{\vec{E}}_{\mathrm{opt\text{-}rf}}(x,z,t)$ we can conclude that $\vec{P}^{(2)}(x,z,t) = \vec{\chi}_{33}^{(2)}(x,z)(\hat{\vec{E}}_{\mathrm{tot}}(x,z,t) \cdot \vec{e}_{\mathrm{DC}}(x,z))^2$ and from here, $\hat{H}_I = \int dV \frac{2}{3}\chi_{33}^{(2)}v_{\mathrm{DC}}(x,z)(\hat{\vec{E}}_{\mathrm{tot}}(x,z,t) \cdot \vec{e}_{\mathrm{DC}}(x,z))^3$. $\cdot$ represents the scalar product. Finally, we find

$$\hat{H}_I = \int dV 2\chi_{33}^{(2)}v_{\mathrm{DC}}(x,z)((\vec{E}_{\mathrm{opt}}(t) + \hat{\vec{E}}_{\mathrm{opt+rf}}(t) + \hat{\vec{E}}_{\mathrm{opt\text{-}rf}}(t)) \cdot \vec{e}_{\mathrm{DC}}(x.z))^2\vec{E}_{\mathrm{rf}}(t) \cdot \vec{e}_{\mathrm{DC}}(x.z). \tag{23}$$

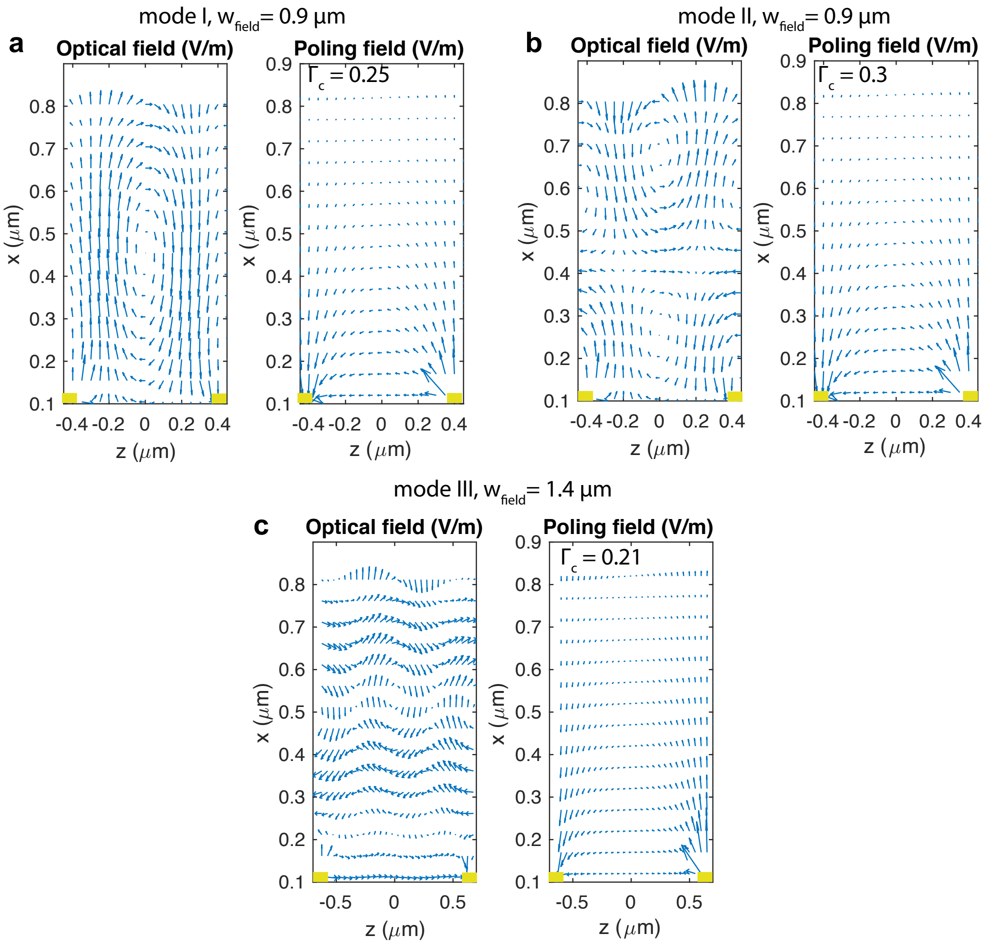

Supplementary figure 1. **Field distribution of optical modes compared to poling fields. a,** mode I, for a $w_{\text{field}} = 0.9\ \mu$m, **b,** mode II, for a $w_{\text{field}} = 0.9\ \mu$m and **c,** mode III, for a $w_{\text{field}} = 1.4\ \mu$m.

As a result, we obtain a total overlap factor that also accounts for the vectorial overlap inside the geometrical dimensions of the organic layer of $A_{per} = w_{\text{field}} \times h_{\text{JRD1}}$:

$$\Gamma_c = \frac{\int_{A_{per}} dA |v_{\text{opt}}(x,z) v_{\text{rf}}(x,z)|^2 |\vec{E}_{\text{opt}}(x,z) \cdot \vec{e}_{\text{DC}}(x,z)|^2}{\int_{\text{everywhere}} dA |v_{\text{opt}}(x,z)|^2} = \frac{A_{\text{overlap}}}{A_{\text{opt}}} \tag{24}$$

We emphasize that the above equation contains, in contrast to eq 8, also the spatial distribution of the nonlinearity as a result of poling. This is the origin of the square dependency on $v_{\text{rf}}(x,z)$ in this equation. The scalar product accounts for the alignment of the polarization of the participating fields with the poling field.

We plot in Supplementary Fig. 1 the field distribution of the three characteristic modes together with the field distribution of the poling fields. From here, we find that for $w_{\text{field}} = 0.9\ \mu$m and $h_{\text{Au}} = 0.03$ nm, $\Gamma_c = 0.25$ for mode I. and $\Gamma_c = 0.3$ for mode II. For $w_{\text{field}} =$

1.4 μm, we find $\Gamma_c = 0.21$ for mode III.

SUPPLEMENTARY NOTE 4. TUNING PROPERTIES OF THE RESONANT MODULATORS

In the following, we analyze the impact of several design parameters on the tuning properties of the modulators.

A. Thickness of metallic stripes

While we show in the main text how the resonances tune with the periodicity w_{field}, we focus our attention on the thickness of the interdigitated electrodes h_{Au}. We restrict our analysis here to mode I at $w_{\text{field}} = 0.9$ μm, but mention that a similar behavior is expected for all resonances studied in our work. Our simulations in Supplementary Fig. 2 show that an increasing h_{Au} leads to a broadening of the guided mode resonance and a slight blue-shift. However, the overlap factor Γ_c does not change dramatically as a function of h_{Au} and is mostly confined in the range [0.3,0.4]. As a result, gold thicknesses up to 70 nm are optimal to insure a high modulation efficiency.

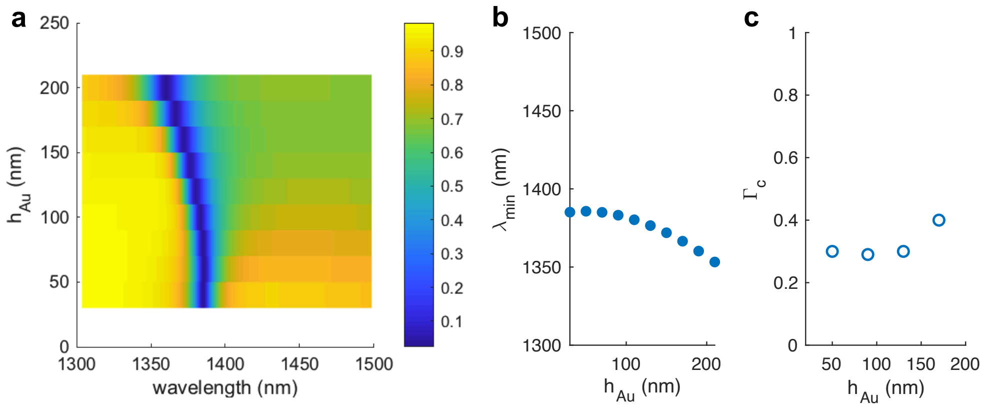

Supplementary figure 2. **Optical properties of the resonant modulators. a,** The resonance linewidth increases as the height of the metallic fingers is increased. **b,** The resonant frequency shifts only slightly. **c,** The overlap factor does not change significantly with h_{Au}.

B. Angle of incidence

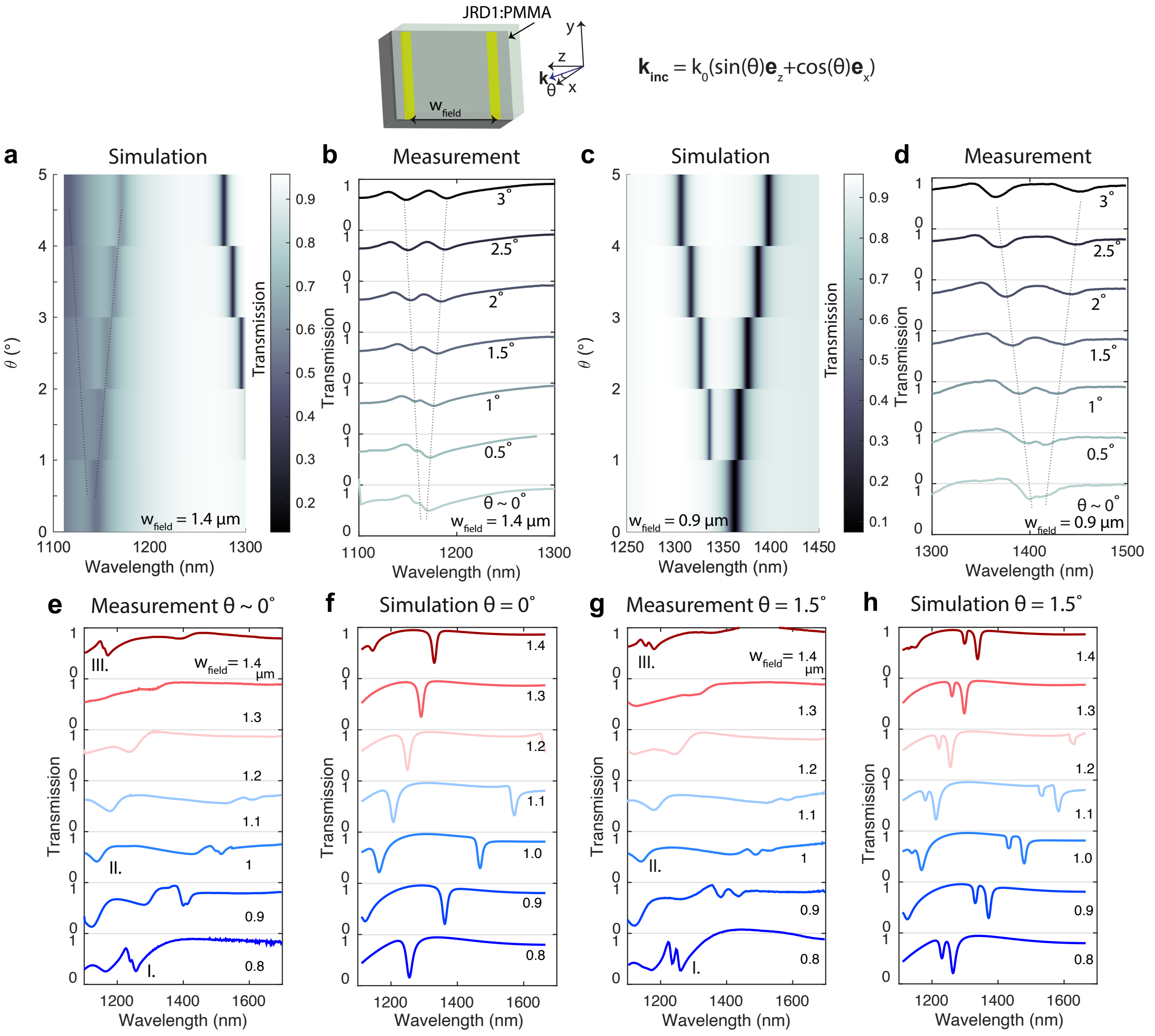

Supplementary figure 3. **Tuning with incident angle θ. a,-d,** At incident angles θ that deviate from normal incidence, a double resonance emerges from a single resonance, characteristic for the guided mode resonances used in this work. We show here exemplarily the example of $w_{\text{field}} = 0.9$ μm and 1.4 μm, the latter being used for the SLMs discussed in the main text. **e,-f,** Measurements and simulations of the transmission for different values of w_{field}, at normal incidence. **g,-h,** Measurements and simulations of the absolute transmission for different values of w_{field}, at an incident angle of $\theta = 1.5°$. Dashed lines mark the tuning of the resonant wavelengths with incident angle.

In Supplementary Fig. 3, we analyse, by experiments and simulation, the dependence

of the transmission on the angle θ of the incident beam, whereas θ is defined with respect to the normal incidence on the yz-plane (see upper panel). In Supplementary Fig. 3 a-d we analyse this behavior exemplarily for two values of $w_{\text{field}} = 0.9\ \mu$m and 1.4 μm. The latter is used in our demonstration of 2 × 2 SLMs discussed in the main text. We find from both simulations and experiments that a double resonance emerges quickly from the single resonance as the angle of incidence starts deviating from the normal. This effect is a characteristic of the guided mode resonances exploited here. In Supplementary Fig. 3 e-h we report the transmission through the sample at $\theta = 0°$ and $\theta = 1.5°$ degrees for various values of w_{field}. We observe that the resonant splitting occurs for all resonances of type I., II. and III. We note that the small splitting that can be observed in the measured spectra of Supplementary Fig. 3 e is attributed to an incident angle that slightly deviates from $\theta = 0°$. On the contrary, we show in Supplementary Fig. 4 that the guided mode resonances are much less sensitive to an incident angle ϕ. This behavior is expected from the translational symmetry of the device along the x-axis.

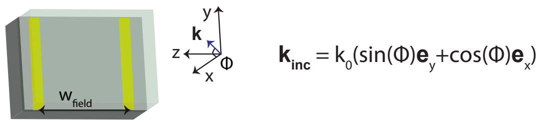

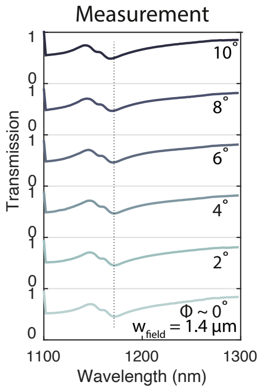

Supplementary figure 4. **Tuning with incident angle ϕ.** Measured transmission spectra at various incident angles ϕ reveal that the guided mode resonances are much less sensitive to this angle over a range of ±10°.

The angular tuning of the resonance discussed above influences the acceptance angle of the SLM while in operation. The operation wavelength λ_{op} of the modulators is typically chosen to coincide with the wavelength of highest slope $\frac{dT}{d\lambda}$, with T being the transmission shown e.g. in Supplementary Fig. 3 b. At this wavelength, the modulation depth $\eta = \frac{T_V - T_0}{T_0}$ is maximal at a given applied voltage. In Supplementary Fig. 5 we show $\frac{dT}{d\lambda}$ for various incident angles onto an SLM (shown in the inset) with $w_{\text{field}} = 1.4\ \mu$m and identify how 1.

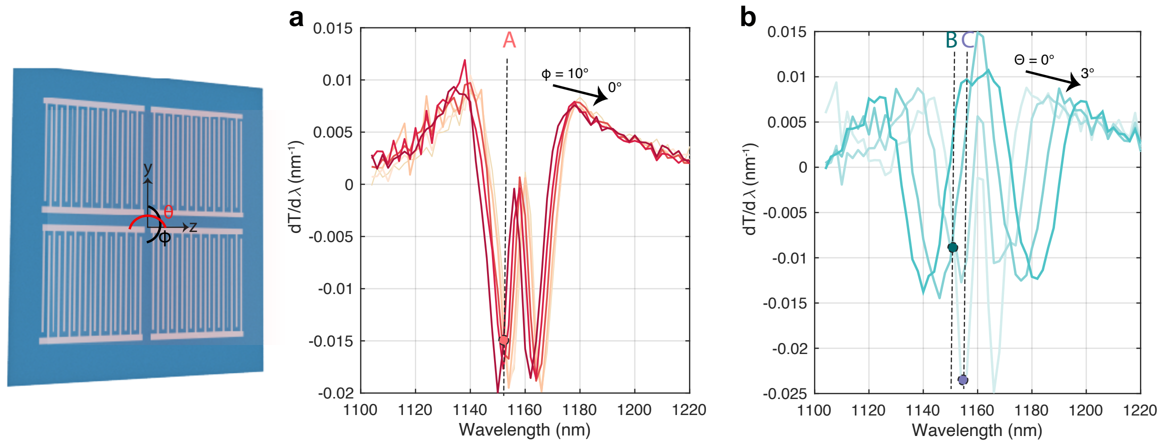

Supplementary figure 5. **Angle-dependent modulation characteristics of SLM at various working operation wavelengths.** The differential change in transmission of the SLM is reported for various incident angles $\phi = 0°, 2°, 4°, 6°, 8°, 10°$ in **a,** and $\theta = 0°, 1°, 2°, 3°$ in **b,**. Point A describes a working point at an operation wavelength where the overall modulation changes only slightly over an incident angle of $\phi = \pm 10°$. At point B, the overall modulation changes only slightly over an incident angle of $\theta = \pm 2°$, but is reduced as compared to the maximum. Point C denotes the working point of maximum modulation at normal incidence, where the modulation rapidly decreases as the incident angle deviates from the normal.

the SLM is little sensitive to the angle ϕ and 2. the SLM is mostly sensitive to the angle θ, since the split of the resonance results into a change in $\frac{dT}{d\lambda}$. For example, we note that at working point A, the modulation depth is approximately constant for the range of angles of $\phi = \pm 10°$. Similarly, at point B, the modulation depth is approximately constant for the range of angles of $\theta = \pm 2°$, albeit at the expense of a modulation depth that is lower than its maximum value. On the contrary, an operation with λ_{op} at the point of maximum slope for normal incidence (working point C) typically results in a narrow acceptance angle, since the modulation depth decreases rapidly as the angle deviates from the normal (e.g.down to 50% loss of modulation depth at $\theta = \pm 1°$). If we define our acceptance angle as the range over which the modulation does not suffer from more than 3 dB loss in magnitude from its maximum value, then we can extract a set of acceptance angles of $\phi = \pm 10°$ and $\theta = \pm 1°$. In general however, in applications that require a wide acceptance angle in conjunction with SLMs that rely on guided mode resonances, a trade-off needs to be made between the combined angle of acceptance (in both ϕ and θ) and modulation depth.

C. Thermo-optical vs. electro-optic modulation

In addition to the electro-optic effect employed for modulation of light in our devices, thermo-optical effects - by which the refractive index of the samples is modulated through a heat-driven process which originates from the applied electrical power (typically below 10 μW) to the samples - can constitute an additional channel for tuning the transmission of an incident beam. Before we proceed to quantify this effect by experiment, it should be noted that there are various characteristics that set apart the electro-optic effect from the thermal effect. First, the electro-optic modulation is, as shown extensively in the main text by theory and measurements, sensitive to the polarity of the applied modulation voltage. This is a direct consequence of the electro-optically introduced refractive index modulation $\Delta n \sim E$. In contrary, a thermo-optical tuning is independent of the polarity of the applied voltage. Second, the electro-optic effect scales linearly with the applied electric field. The thermo-optical effect is expected to scale non-linearly, owing to the diode-shaped current-voltage characteristics (see Fig. 2b of the main text) of these devices, and is expected to become increasingly dominant at high applied voltages. The presented modulation data in Fig. 4 of the main text clearly exhibit all characteristics of the electro-optic effect. Nevertheless, we show in Supplementary Fig. 6 experimental data that quantifies the thermo-optical effect for the case of $w_{\text{field}} = 0.9$ μm (measured transmission reported in Supplementary Fig. 3 d), and at an applied voltage of 40 V, corresponding to an applied field of 57.1 Vμm^{-1}. To quantify the thermo-optical effect alone, we perform a comparative analysis of the modulation efficiency of a sample prior to and after its activation by electric field poling. In the former case, the electro-optic effect is vanishingly small and only thermal effects become visible. We investigate the sample at a modulation speed of 100 kHz and 1 MHz, which are in the range of interest for the SLMs discussed here. We find from Supplementary Fig. 6 a and b that indeed thermal effects lead to a tuning behavior which is - as expected - independent of the polarity of the applied voltage. Moreover, its magnitude is negligible (at 40 V and 100 kHz is as low as 0.3 % for mode I. and 0.5 % for mode II. and at 1 MHz 0.25 % for mode I. and 0.35 %) compared to the electro-optic effect (at 40 V and 1 MHz 8 % for mode I. and 16 % for mode II.), as can be noticed from comparing Supplementary Fig. 6 b and c.

Recent advances in the development of electro-optic organic compounds [4, 5], as well as previously demonstrated approaches to realize current injection barriers from benzocy-

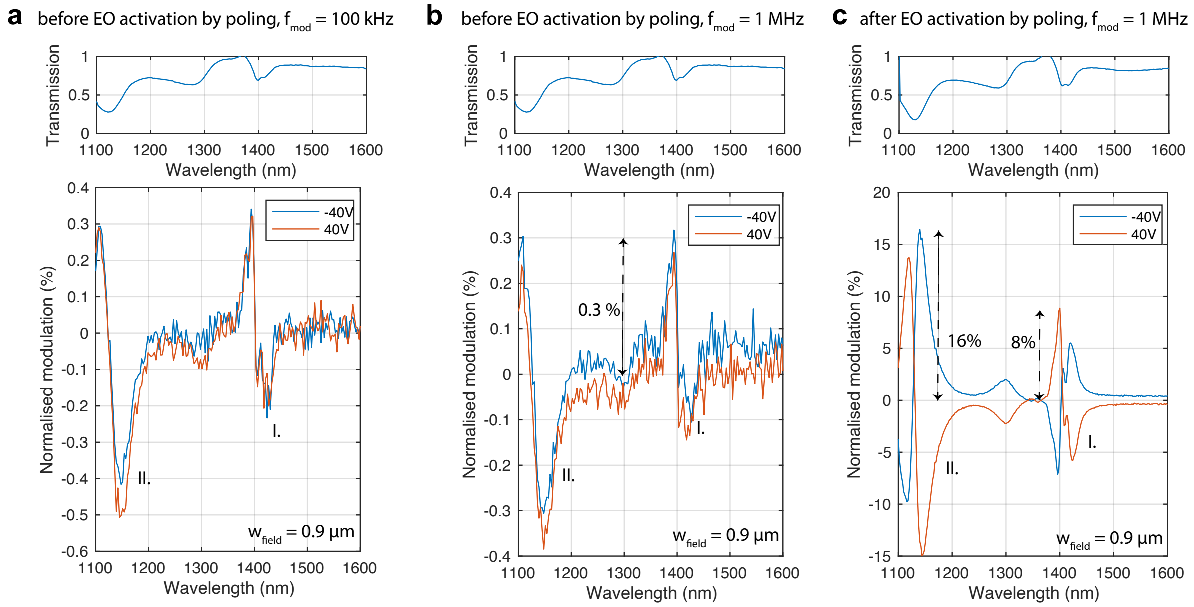

Supplementary figure 6. **Measured thermo-optical vs electro-optic modulation.** The modulation depth η is compared for the same sample prior to and after electro-optic activation by poling (respective transmission curves shown in the upper graphs). For an unpoled sample shown in **a,-b,** , the found modulation depth is η = 0.3 % for mode I. and η = 0.5 % for mode II. at 100 kHz modulation speed, and η = 0.25 % for mode I. and η = 0.35 % for mode II. at 1 MHz. The polarity of the applied voltage has no influence on the modulation, in contrast to the electro-optic effect shown in **c,**, which introduces a much higher modulation depth of η = 8 % for mode I. and η = 16 % for mode II. at 1 MHz.

clobutene or titanium dioxide [2] have the potential to lead to electro-optic devices with thermal and electro-optic properties beyond the ones discussed in this study.

D. Linewidth characteristics of the probing laser

In our experiments, we use a tunable laser (super-K Select) to probe the modulation characteristics of our modulators. In Supplementary Fig. 7 we plot the measured spectra and full-width half maximum of its emission throughout the NIR, which exceeds 4 nm and can range up to 11.5 nm. The ability of our modulators to introduce significant intensity modulation even to a broadband incident beam are an experimental proof that the modulators are - in principle - compatible with compact laser diodes, which have relatively broad

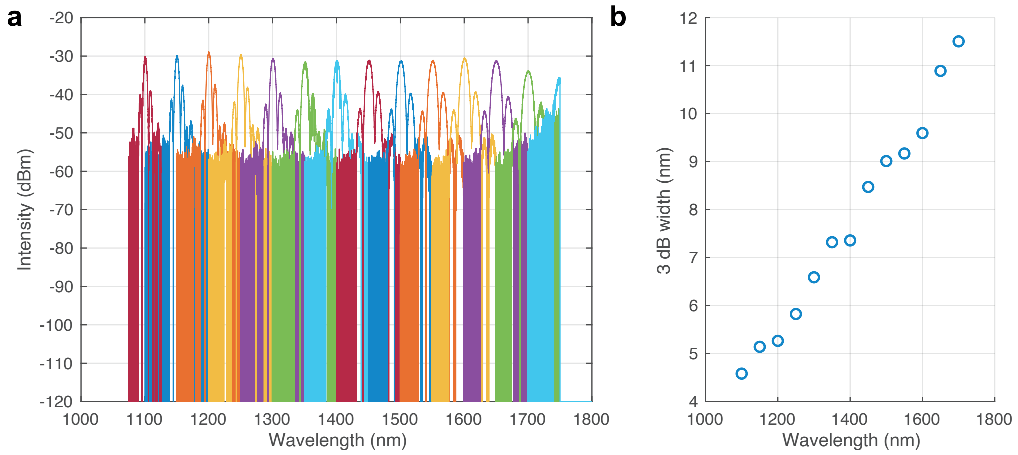

Supplementary figure 7. **Linewidth of the probing laser. a,** spectra measured with an optical spectrum analyser **b,** 3-dB linewidth as a function of centre wavelength.

linewidths. Even more, they can accommodate wavelength tolerances in the fabrication of laser diodes which are typically up to 10 nm (e.g. Eblana Photonics EP1310-ADF-B laser diode). This property clearly outperforms any prior demonstrations of electro-optic metasurfaces which trade a very specific and narrow operation point for high intensity/phase tuning properties. Overall, all these properties render the electro-optic free-space modulators presented here compatible with real-life applications that have stringent requirements regarding their compactness and power dissipation.

E. Discussion about overlap factor and possible routes to improve performance and expand applications in the future

In this work, we exploit the electro-optic effect in combination with resonant guided modes to introduce an intensity modulation to an incident beam. As shown above, the refractive index of the active molecules changes as $\Delta n(t) = -\frac{1}{2}n_{\mathrm{mat}}^3 r_{33} E_{\mathrm{rf}}(t)$ and effects a shift of the resonant frequency as follows $\frac{\Delta\lambda_{\mathrm{res}}}{\lambda_{\mathrm{res}}} = -\frac{\Delta\omega_{\mathrm{res}}(t)}{\omega_{\mathrm{res}}} = \frac{\Delta n(t)}{n_{\mathrm{mat}}}\Gamma_c$. We identify three distinct aspects that influence the modulation efficiency $\eta = \frac{\Delta T}{T_0}$ of the resonant modulators discussed in this work: first, the overlap Γ_c between the optical, RF fields and the JRD1 film; second, the electro-optic coefficient r_{33} of the active layer; and third, the linewidth of the resonance $\delta\omega$ and/or the probing laser $\delta\omega_{\mathrm{laser}}$.

For guided mode resonance of the type discussed here we find an overlap factor on the

order of $\Gamma_c = 0.25$ and an in-device electro-optic coefficient of $r_{33} = 105$ pm/V. These values exemplify that using traditional electro-optic materials which have typically 4-100 times lower r_{33} with the same simple geometry would result in much lower electro-optic frequency shift $\Delta\omega_{\mathrm{res}}$. Furthermore, the in-plane periodic poling of the film demonstrated here is essential to achieve a refractive index change that is equal in between all metallic fingers of the interdigitated array, and this might need to be achieved for traditional materials as well. These characteristics underlines the potential that electro-optic molecules might have on active free-space photonics. Importantly, alternative geometries that increase the overlap of the optical mode with the active material towards $\Gamma_c = 1$ could benefit a larger variety of applications in the future. To that end, resonant structures may be considered that exhibit highly localized modes. Examples could be surface plasmon resonances that were experimentally demonstrated using AlN (has an $r_{33} = 1$ pm/V) discussed in Ref. [6] or microcavity arrays proposed theoretically in Ref. [7]. Achieving such high overlap factors could lead to an increase of the tuning range beyond $\Delta\lambda_{\mathrm{res}} = 11$ nm demonstrated here to few tens of nanometers. Clearly, several additional challenges will need to be addressed regarding the fabrication of such devices, but our proposal to apply the film post-fabrication by spin-coating might be a promising path in the future.

The linewidth of the resonance employed for the electro-optic modulation is important in real-life settings. For example, free-space electro-optic modulators will possibly need to achieve high modulation performance even for cheap laser diodes which have typically a linewidth of few nanometers, rather than laboratory-based narrow-band lasers. Consequently, it is instrumental to achieve a wavelength shift larger than the resonance linewidth, that is in turn larger than the laser linewidth $\Delta\omega_{\mathrm{res}} > \delta\omega > \delta\omega_{\mathrm{laser}}$. The tuning performance we demonstrate ($\Delta\lambda_{\mathrm{res}} = 11$ nm tuning at $\lambda_{\mathrm{res}} = 1400$ nm) determines the current bounds that can be achieved with JRD1:PMMA, but also in this context, surface plasmon resonances discussed in Ref. [6] that have higher quality factors may achieve full intensity suppression at low applied voltages.

F. Equivalent circuit of high-frequency modulation

In this section, we discuss the theoretical high-frequency cut-off and compare it to the experimental results. We start by estimating the theoretical cut-off of the active part of our

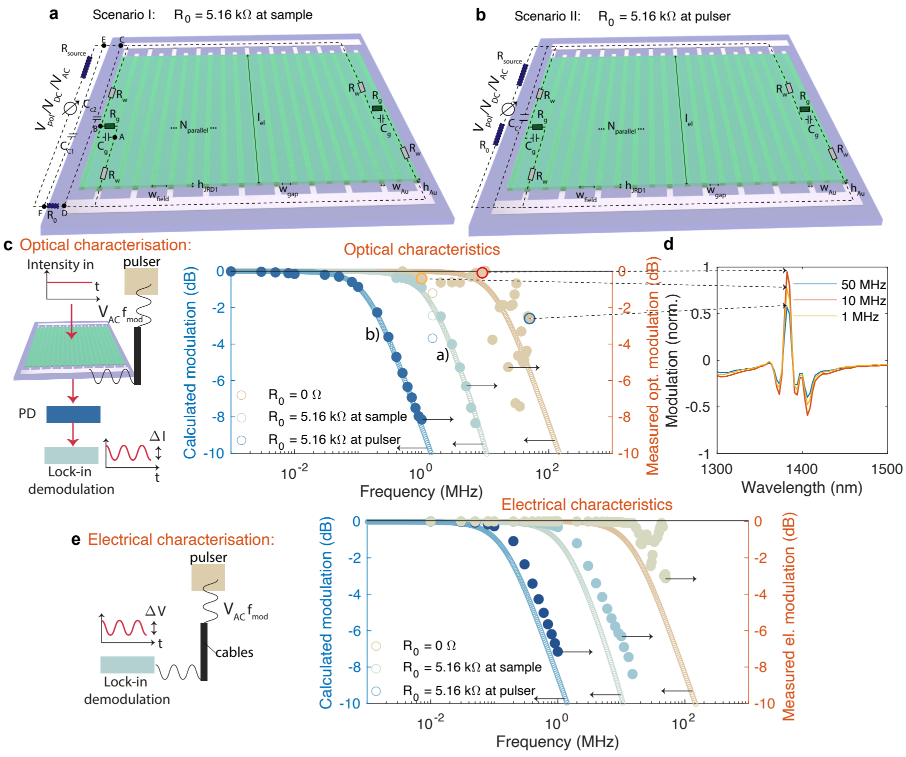

Supplementary figure 8. High-frequency modulation. a,-b, Equivalent circuit of a single electro-optic transducer to which we connect an optional series resistor R_0. We investigate two scenarios: I. the resistor is connected as close as possible to the sample and II. the resistor is connected directly after the pulser. c, Measured intensity modulation of the optical beam is compared to the expected modulation amplitude found from the equivalent circuit. Clearly, the cut-off depends on the value and the position of R_0 prior to the sample, thereby underlining that the main cut-off originates from the cabling prior to the sample. d, Measured wavelength-resolved intensity modulation is provided for three modulation frequencies. At $f_{\mathrm{mod}} = 50$ MHz, the maximal modulation is about 50% of its maximal value, thereby determining the current demonstrated 3-dB bandwidth of the modulators discussed here. e, Additionally, the system formed by the pulser and cabling is characterized electronically. The voltage at the sample port is monitored directly with a lock-in amplifier and we find it to depend similarly like in c, on the location and magnitude of R_0. We conclude that the RC time constant is currently limited by combined pulser-cabling system.

modulators, and consider for the moment the ideal case where the cables used to apply the pulses to the modulator have a negligible capacitance, and $C_{\mathrm{C1}} = 0$ nF and $C_{\mathrm{C2}} = 0$ nF. The high-frequency behavior of a single electro-optic transducer can be modeled using an

equivalent circuit as shown in Supplementary Fig. 8 a. R_w is the resistance of one single metallic wire of length l_{el}, width w_{Au} and height h_{Au}. R_{g} is the resistance in the gap between a single pair of metallic wires and has been retrieved from the current-voltage curve shown in the main text. C_{g} is the capacitance between one single pair of metallic wires of distance w_{gap}. R_0 is an externally loaded series resistor that is added to the internal resistance of the source ($R_{\text{source}} = 50\ \Omega$) for the modulation speed experiments [8]. In all other studies, $R_0 = 0\ \Omega$. Given that the interdigitated array is made from N_{parallel} periods of mutual interdigitated finger pairs, we find that the impedance across the gap is $Z_{\text{AB}} = Z_{\text{g}} = \frac{R_{\text{g}}}{1+j\omega C_{\text{g}} R_{\text{g}}}$, the impedance in one single pair of metallic wires is $Z_{\text{single pair}} = 2R_w + \frac{R_{\text{g}}}{1+j\omega C_{\text{g}} R_{\text{g}}}$, the impedance of all N_{parallel} interdigitated finger pairs is $Z_{\text{interdigitated}} = \frac{Z_{\text{single pair}}}{N_{\text{parallel}}}$ and the total impedance of the entire circuit is $Z_{\text{total}} = R_0 + R_{\text{source}} + Z_{\text{interdigitated}}$. The high-frequency modulation across a single gap can be retrieved by computing the voltage V_{g} across the gap as a function of applied external voltage V_{AC} and the frequency-dependent impedance:

$$V_{\text{g}} = \frac{Z_{\text{g}}}{Z_{\text{single pair}}} \frac{Z_{\text{interdigitated}}}{Z_{\text{total}}} V_{\text{AC}}. \tag{25}$$

Using the formula above, the high-frequency modulation cut-off can be analytically computed for typical values of $R_w = 690\ \Omega$, $C_{\text{g}} = 0.47 \times 10^{-15}$ F, $R_{\text{g}} = 3 \times 10^{11}\ \Omega$ and $N_{\text{parallel}} = 300$. We find that the 3-dB cut-off frequency of the active part of the sample is 13.1 GHz if an external load of resistance $R_0 = 0\ \Omega$ is connected to the circuit (unloaded circuit). Also, for the specific case where $R_w \to 0$ and $R_{\text{g}} \to \infty$ compared to all other resistances in the system, we can approximate that

$$V_{\text{g}} = \frac{1}{1 + j\omega C_{\text{g}} N_{\text{parallel}} (R_0 + R_{\text{source}})} V_{\text{AC}}. \tag{26}$$

As expected, the cut-off frequency is inversely proportional to the capacitance in the gap C_{g} and the sum between the externally loaded resistor R_0 and the source resistance R_{source}. Clearly, the cut-off frequency is expected to increase proportionally for smaller pixels and will enable even higher modulation speeds. The low capacitance C_{g} is strictly connected to the interdigitated array architecture we chose and is in principle favorable compared to a parallel plate geometry.

In our real system however, the sample is connected via coaxial cables to the pulser that provides the modulating pulses, which we will show in the following to dominate the

frequency cut-off of our measurements, an effect which is particularly strong when the series load resistance is high and dominate over inductive impedances from the coaxial cables. Typical coaxial cables have capacitances on the order of 96 pF/m. Considering that cables as long as 2.25 m are necessary in our concrete experimental implementation to reach the sample, the cable capacitance can be estimated to be above $C_C = 19.44$ nF, and will be, compared to all other capacitances in the system (including the sample capacitance) the one that dominates the cut-off of the combined pulser-sample system. We demonstrate this fact experimentally by measuring both the optical and the electrical cut-off frequency in two distinct scenarios, shown in Supplementary Fig. 8 a and b. To this end, we attach an external series resistance of $R_0 = 5.16\ k\Omega$ as close as possible to the sample (a,) and as close as possible to the pulser (b,). Consequently, in this case we can write that $Z_{\mathrm{CD}} = \frac{1}{j\omega C_{\mathrm{C2}}+1/Z_{\mathrm{interdigitated}}}$ and $Z_{\mathrm{EF}} = \frac{1}{j\omega C_{\mathrm{C1}}+\frac{1}{R_0+Z_{\mathrm{CD}}}} = \frac{R_0+Z_{\mathrm{CD}}}{j\omega C_{\mathrm{C1}}(R_0+Z_{\mathrm{CD}})+1}$, with C, D, E and F the points as marked in the figure. Using these definitions, we now find that the equivalent circuit can be modeled as:

$$V_{\mathrm{g}} = \frac{Z_{\mathrm{g}}}{Z_{\text{single pair}}} \frac{Z_{\mathrm{CD}}}{Z_{\mathrm{CD}} + R_0} \frac{Z_{\mathrm{EF}}}{Z_{\mathrm{EF}} + R_{\mathrm{source}}} V_{\mathrm{AC}}. \tag{27}$$

Given the formula above, we now estimate that in the scenario discussed in a), the circuit capacitances are $C_{\mathrm{C1}} = 19.2$ nF and $C_{\mathrm{C2}} = 0.24$ nF and in the scenario discussed in b) $C_{\mathrm{C1}} = 0$ nF and $C_{\mathrm{C2}} = 19.44$ nF. We report the frequency-dependent behavior we find with this formula with the hollow circles in Supplementary Fig. 8 c. We clearly find that the cut-off depends not only on the presence of the external resistor but also on the location where the external resistor is inserted into the circuit prior to the sample (close to sample vs close to pulser). Consequently, we can conclude that the cut-off is mostly limited by the system rather than the sample. To confirm this, we further compare this expected behavior to our experiments. In Supplementary Fig. 8 c, we report in solid dots the modulation of the optical signal that traverses our modulator and is detected by a photodiode (as shown in left panel). We find a 3-dB cut-off frequency of 50 MHz in the case of the unloaded circuit, further confirmed by the wavelength-dependent modulation reported in Supplementary Fig. 8 d at three characteristic modulation frequencies. Furthermore we find, as expected, that the cut-off of the optical modulation cut-off is decreased if the external circuit is loaded with R_0, and that it depends strongly on the location at which this external resistor is inserted. In a second step, we investigate the pure electrical cut-off of the signal that is applied to our modulators, with the goal to characterise the system prior to the

sample itself. To this end, instead of applying the driving voltage to the sample, we use the same cable configuration to feed the signal directly into the lock-in amplifier (AC coupled) as shown in Supplementary Fig. 8 e. This analysis allows us to characterise the pulses that reach the modulator in the first place and thus trigger the electro-optic effect. The results of the electrical characterisation are reported in Supplementary Fig. 8 e. We find clearly that, owing to the external circuit, the pulses are strongly attenuated at the port of the modulators, and that the cut-off has a similar dependency on the position and presence of the external resistor R_0. Interestingly, we find that even in the case of the unloaded circuit, at a modulation frequency of 50 MHz, we can inject only 50 % of the modulating voltage into the port of the sample, thereby suggesting that the actual cut-off of the sample might be higher than experimentally demonstrated in this work. However, to confirm this possibility, further studies are necessary in the future along with further improvements to the current system, including high-frequency compatible cables and probes, high-frequency driving voltages and a matched RF circuit on-chip.

SUPPLEMENTARY NOTE 5. MULTI-COLOR SLMS

We harness the broadband electro-optic effect to realize a proof of concept multi-color SLM that consists of five individual pixels connected and activated in parallel, as shown in Supplementary Fig. 9 a that span the frequency range from 1530 nm to 1630 nm as shown in Supplementary Fig. 9 b by design of the periodicity of the metallic grating $w_{gap} = 0.8$, 0.82, 0.84, 0.86, and 0.88 μm. We show in the appended video a demonstration of how the individual pixels, while connected in parallel to the same actuating voltage of $V_{\text{peak}} = 40$ V at $f_{\text{mod}} = 2$ Hz, modulate an incident light only when its wavelength is near the resonant frequency of the individual pixels. The video shows a sequence of five raw image stacks acquired directly with the InGaAs camera when the entire SLM is illuminated by a Santec laser beam (linewidth 200 kHz) at the working points indicated by the black dots. This demonstrates how multi-color pixels can be straightforwardly combined into one single device to achieve much more sophisticated color-dependent modulation that spans a wide wavelength range.

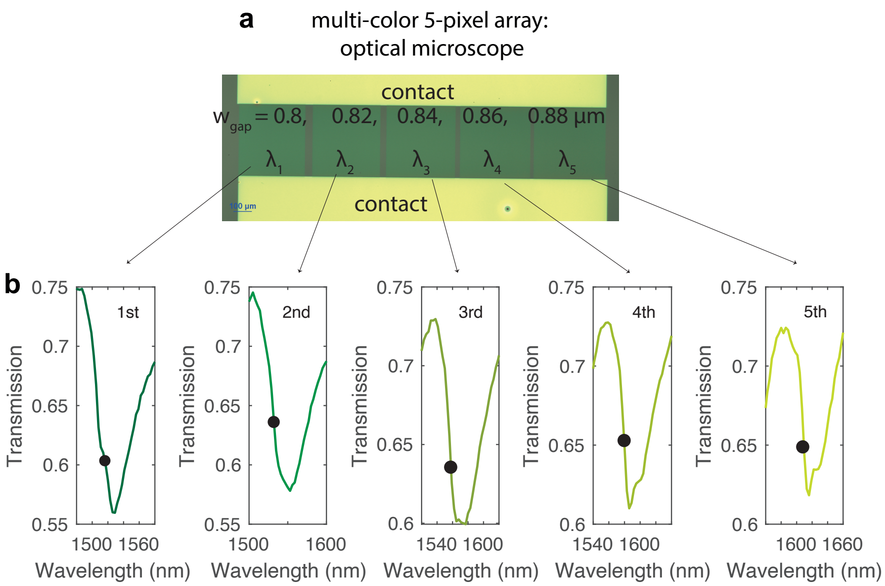

Supplementary figure 9. **Multi-color SLM. a,** five pixels with distinct resonant wavelength $\lambda_1 - \lambda_5$ as shown in **b,** are assembled into a single multi-color SLM and activated and operated concomitantly, in parallel. The black dots represent the working points for the image stacks combined into an animated gif attached to the paper.

SUPPLEMENTARY NOTE 6. NUMERICAL CALCULATIONS

The optical response of the spatial light modulator was simulated using the frequency domain finite element Maxwell solver in CST Microwave Studio Suite 2020. The JRD1:PMMA film is modeled as a homogenous film of height $h_{\mathrm{JRD1}} = 690$ nm with isotropic real and imaginary dielectric function $\epsilon_{\mathrm{JRD1}}(\lambda) = \epsilon_{\mathrm{r}}(\lambda) + i\epsilon_{\mathrm{i}}(\lambda)$ as shown in Fig. 2 d in the main text. Additionally, we provide in Supplementary Fig. 10 the wavelength-dependent dielectric function of the JRD1:PMMA mixture for various concentrations for interested readers. The complex refractive index is measured using variable angle spectroscopic ellipsometry (VASE) on neat, unpoled films deposited on glass substrates. The quartz substrate is only little dispersive over the band of interest from 1100 nm to 1600 nm and is hence modeled with an $\epsilon_{SiO2} = 2.0736$. The interdigitated array of gold electrodes is modeled as a lossy metal with parameters as given in the library of CST Microwave Studio Suite (electric conductivity of $\sigma_{\mathrm{Au}} = 4.5 \times 10^7$ S/m).

The transverse electric field distribution of the static tuning field shown in Supplementary Fig. 1 was simulated using the Electrostatic Solver in CST Microwave Studio Suite 2020. In

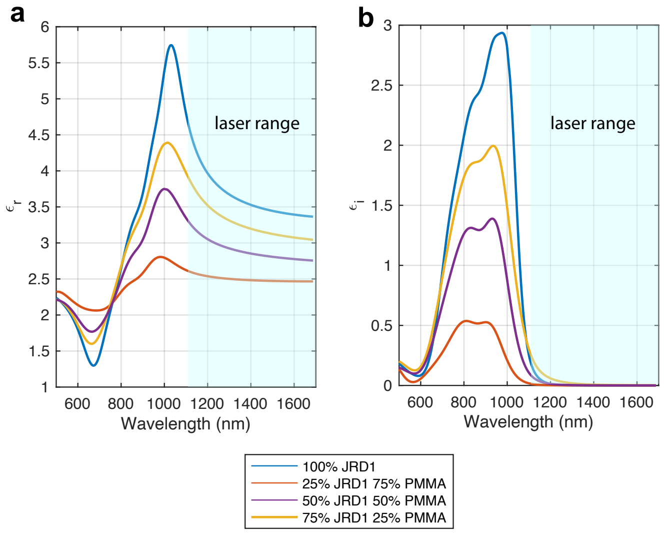

Supplementary figure 10. **Complex dielectric function of JRD1:PMMA mixture for various concentrations in the wavelength range from 500 nm to 1700 nm. a,-b,** Real and imaginary parts of $\epsilon_{\mathrm{JRD1}}(\lambda)$ demonstrate the engineered absorption in the red part of the visible spectrum and a high index of refraction and low losses at telecom wavelengths [2].

these simulations, the JRD1:PMMA film was assumed to have a height of $h_{\mathrm{JRD1}} = 690$ nm and a dielectric constant of $\epsilon_{\mathrm{JRD1:PMMA}} = 5$. The metallic array is modeled as a perfect electric conductor. The quartz substrate is assumed to have a dielectric constant of $\epsilon_{SiO_2} = 3.75$ at zero frequency.

SUPPLEMENTARY NOTE 7. WAVELENGTH-RESOLVED CHANGE IN REFRACTIVE INDEX

In this section we demonstrate experimentally the broadband non-linearity of the JRD1:PMMA mixture. In Supplementary Fig. 11 a-b we provide the refractive index change of a film of 50%wt JRD1:PMMA as a function of applied voltage, measured by ellipsometry on a vertical stack test sample as shown in the inset. The height of the JRD1:PMMA layer was 1.85 μm. On this sample, we measured an electro-optic coefficient $r_{33} = 100$ pmV^{-1} at 1310 nm by Man-Teng ellipsometery [9]. As expected, we find that the relative refractive index change $\frac{\Delta n}{n} = \frac{n(E_{\mathrm{DC}})-n(0)}{n(0)}$ scales approximately linearly with the applied electric field. $n(E_{\mathrm{DC}})$ is the refractive index at an arbitrary applied electric field E_{DC} and $n(0)$ is

the refractive index at $E_{\text{field}} = 0$. Small deviations from a linear dependency can appear as a result of the recursive fitting procedure we employed to retrieve the refractive index data from the ellipsometric measurements of the vertical stack. Moreover, $\frac{\Delta n}{n}$ changes only slightly in the band from 1200 nm to 1500 nm and decays quicker below 1200 nm.

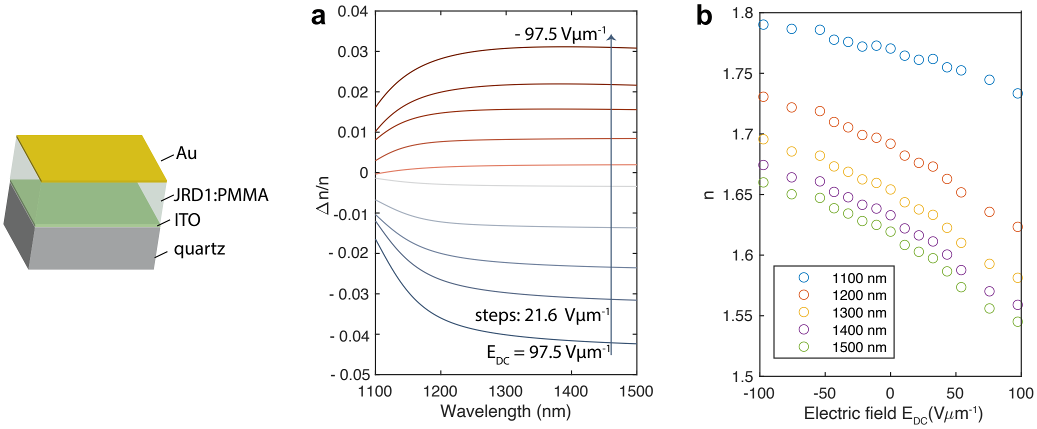

Supplementary figure 11. **Wavelength-resolved change in refractive index of JRD1:PMMA with applied bias field E_{DC}.** **a,** Refractive index change upon various applied fields show an approximately flat relative change in refractive index over the range from 1200 nm to 1500 nm, over a field swing from 97.5 Vμm^{-1} to -97.5 Vμm^{-1}. **b,** The refractive index n is plotted for several wavelengths as a function of applied electric field.

SUPPLEMENTARY NOTE 8. SAMPLE DETAILS

Below we characterize the properties of our samples using scanning electron microscopy (SEM), atomic force microscopy (AFM) and optical microscopy (BF = bright field and DF = dark field).

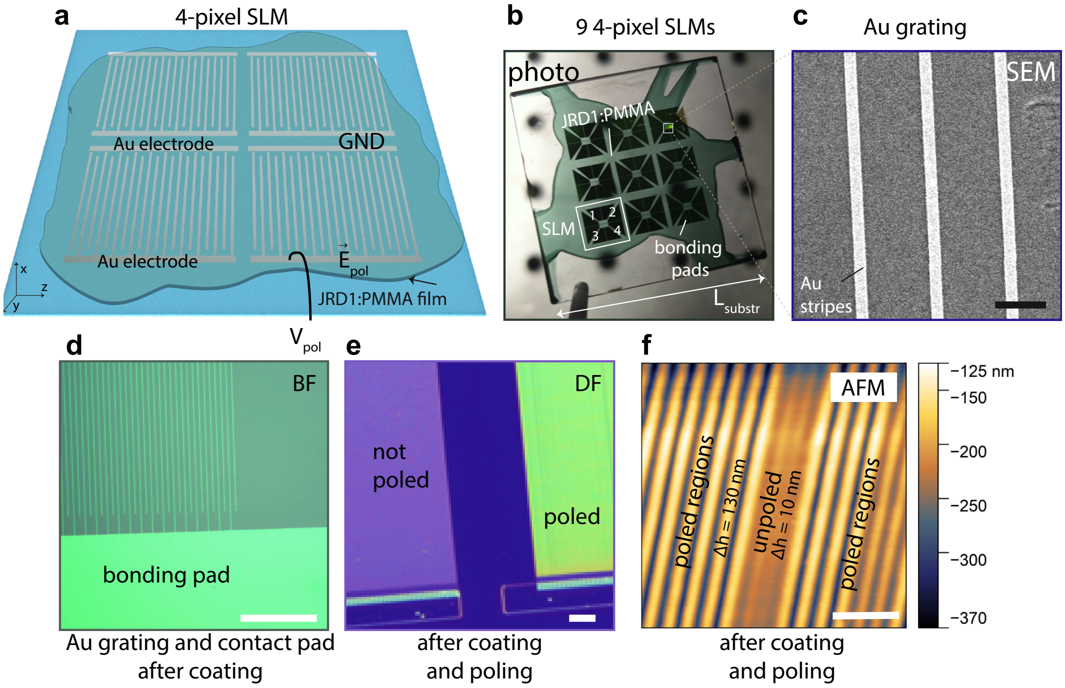

Supplementary figure 12. **Properties of the resonant electro-optic SLM a,** A single SLM is formed from four electro-optic modulators patterned in a 2×2 matrix on a quartz substrate that is covered by a single layer of nonlinear electro-optic molecules JRD1 mixed with PMMA. The metallic structures are contacted with the two electrodes used to apply a poling and a drive voltage to the modulator. **b,** Picture shows multiple modulator arrays and their corresponding pads 1-4 assembled into 4-pixel SLMs. On one substrate of dimensions L_{substr} equal to one inch we pack a total of 9 SLMs. The green coating is the JRD1:PMMA layer. The substrate dimension L_{substr} is equal to one inch. **c,** Scanning electron micrograph of the fabricated devices prior to coating shows high-quality metallic stripes of a width of 200 nm. Scale bar = 1 μm. **d,** Optical microscope image in bright field mode confirms excellent film formation after deposition with no visible inhomogeneities. Scale bar = 10 μm. **e,** Poled devices are inspected by optical microscope imaging in dark field mode, where a clear contrast in scattering amplitude can be observed from the poled device as compared to the not poled device. Scale bar = 20 μm. **f,** Atomic force microscopy measurements reveal a consistent and uniform change in topography of the organic film after poling, demonstrated by a device which contains purposefully both poled and unpoled regions for direct comparison. The poled regions exhibit relief changes on the order of Δh = 130 nm and unpoled regions on the order of Δh = 10 nm, thereby confirming a homogenous film after deposition. Scale bar = 10 μm. SLM = spatial light modulator, BF = bright field, DF = dark field, GND = ground, AFM = atomic force microscopy.

SUPPLEMENTARY REFERENCES

[1] Ileana-Cristina Benea-Chelmus, Yannick Salamin, Francesca Fabiana Settembrini, Yuriy Fedoryshyn, Wolfgang Heni, Delwin L Elder, Larry R Dalton, Juerg Leuthold, and Jérôme Faist, "Electro-optic interface for ultrasensitive intracavity electric field measurements at microwave and terahertz frequencies," Optica **7**, 498–505 (2020).

[2] Wenwei Jin, Peter V Johnston, Delwin L Elder, Andreas F Tillack, Benjamin C Olbricht, Jinsheng Song, Philip J Reid, Ruimin Xu, Bruce H Robinson, and Larry R Dalton, "Benzocyclobutene barrier layer for suppressing conductance in nonlinear optical devices during electric field poling," Applied Physics Letters **104**, 243304 (2014).

[3] Delwin L Elder, Stephanie J Benight, Jinsheng Song, Bruce H Robinson, and Larry R Dalton, "Matrix-Assisted Poling of Monolithic Bridge-Disubstituted Organic NLO Chromophores," Chemistry of Materials **26**, 872–874 (2014).

[4] Huajun Xu, Lewis E Johnson, Yovan de Coene, Delwin L Elder, Scott. R Hammond, Koen Clays, Larry R Dalton, and Bruce H Robinson, "Bis(4-dialkylaminophenyl)heteroarylamino donor chromophores exhibiting exceptional hyperpolarizabilities," J. Mater. Chem. C **9**, 2721–2728 (2021).

[5] Huajun Xu, Fenggang Liu, Delwin L Elder, Lewis E Johnson, Yovan de Coene, Koen Clays, Bruce H Robinson, and Larry R Dalton, "Ultrahigh Electro-Optic Coefficients, High Index of Refraction, and Long-Term Stability from Diels–Alder Cross-Linkable Binary Molecular Glasses," Chemistry of Materials **32**, 1408–1421 (2020).

[6] Alexei Smolyaninov, Abdelkrim El Amili, Felipe Vallini, Steve Pappert, and Yeshaiahu Fainman, "Programmable plasmonic phase modulation of free-space wavefronts at gigahertz rates," Nature Photonics **13**, 431–435 (2019).

[7] Cheng Peng, Ryan Hamerly, Mohammad Soltani, and Dirk R Englund, "Design of high-speed phase-only spatial light modulators with two-dimensional tunable microcavity arrays," Opt. Express **27**, 30669–30680 (2019).

[8] Hou-Tong Chen, Sabarni Palit, Talmage Tyler, Christopher M Bingham, Joshua M O Zide, John F O'Hara, David R Smith, Arthur C Gossard, Richard D Averitt, Willie J Padilla, Nan M

Jokerst, and Antoinette J Taylor, “Hybrid metamaterials enable fast electrical modulation of freely propagating terahertz waves,” Applied Physics Letters **93**, 91117 (2008).

[9] C C Teng and H T Man, “Simple reflection technique for measuring the electro-optic coefficient of poled polymers,” Applied Physics Letters **56**, 1734–1736 (1990).
