# Supplementary material for: Electro-optic spatial light modulator from an engineered organic layer
Source: Nat Commun. 2021 Oct 11;12:5928. doi: 10.1038/s41467-021-26035-y (PMC8505481; doi:10.1038/s41467-021-26035-y)
Supplement: Supplementary file 2 — Description of Additional Supplementary Files [file 41467_2021_26035_MOESM2_ESM.pdf]

Description of Additional Supplementary Files

File name: Supplementary Movie 1
Description: Raw stack of images of transmitted light through the multi-color spatial light modulators while the laser central wavelength is tuned into resonance with the individual pixels subsequently.
