# Supplementary figures and images for: Electro-optic spatial light modulator from an engineered organic layer
Source: Nat Commun. 2021 Oct 11;12:5928. doi: 10.1038/s41467-021-26035-y (PMC8505481; doi:10.1038/s41467-021-26035-y)

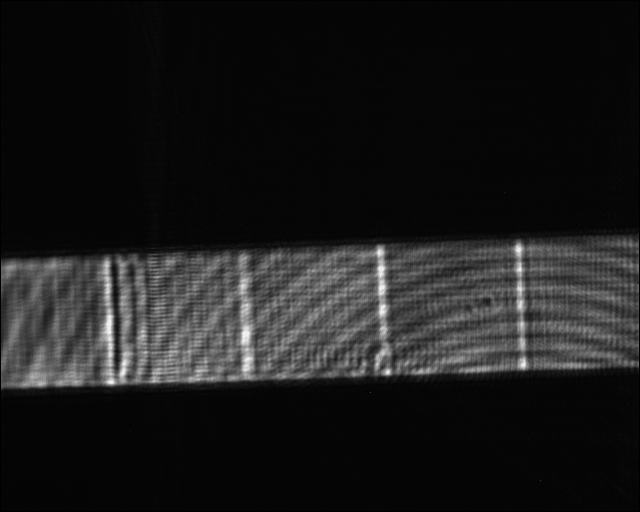

Supplement: Supplementary file 3 — Supplementary Movie 1 [file 41467_2021_26035_MOESM3_ESM.gif]
